# Prolactin receptor signaling induces acquisition of chemoresistance and reduces clonogenicity in acute myeloid leukemia

**DOI:** 10.1186/s12935-023-02944-4

**Published:** 2023-05-19

**Authors:** Laia Cuesta-Casanovas, Jennifer Delgado-Martínez, Josep M. Cornet-Masana, José M. Carbó, Antònia Banús-Mulet, Francesca Guijarro, Jordi Esteve, Ruth M. Risueño

**Affiliations:** 1 Josep Carreras Leukaemia Research Institute (IJC), Campus ICO-GTP, Crta Can Ruti, Camí de les Escoles, s/n, 08916 Badalona, Barcelona, Spain; 2 Faculty of Biosciences, Autonomous University of Barcelona, Barcelona, Spain; 3 Faculty of Pharmacy, University of Barcelona, Barcelona, Spain; 4 Department of Hematology, Hospital Clínic, Barcelona, Spain; 5 Faculty of Medicine, University of Barcelona, Barcelona, Spain; 6 Institut d’Investigacions Biomèdiques August Pi i Sunyer (IDIBAPS), Barcelona, Spain

**Keywords:** Prolactin receptor, Leukemia, Senescence, Biomarker, Differentiation

## Abstract

**Background:**

Development of precision medicine requires the identification of easily detectable and druggable biomarkers. Despite recent targeted drug approvals, prognosis of acute myeloid leukemia (AML) patients needs to be greatly improved, as relapse and refractory disease are still difficult to manage. Thus, new therapeutic approaches are needed. Based on in silico-generated preliminary data and the literature, the role of the prolactin (PRL)-mediated signaling was interrogated in AML.

**Methods:**

Protein expression and cell viability were determined by flow cytometry. Repopulation capacity was studied in murine xenotransplantation assays. Gene expression was measured by qPCR and luciferase-reporters. SA-β-Gal staining was used as a senescence marker.

**Results:**

The prolactin receptor (PRLR) was upregulated in AML cells, as compared to their healthy counterpart. The genetic and molecular inhibition of this receptor reduced the colony-forming potential. Disruption of the PRLR signaling, either using a mutant PRL or a dominant-negative isoform of PRLR, reduced the leukemia burden in vivo, in xenotransplantation assays. The expression levels of PRLR directly correlated with resistance to cytarabine. Indeed, acquired cytarabine resistance was accompanied with the induction of PRLR surface expression. The signaling associated to PRLR in AML was mainly mediated by Stat5, in contrast to the residual function of Stat3. In concordance, Stat5 mRNA was significantly overexpressed at mRNA levels in relapse AML samples. A senescence-like phenotype, measured by SA-β-gal staining, was induced upon enforced expression of PRLR in AML cells, partially dependent on ATR. Similar to the previously described chemoresistance-induced senescence in AML, no cell cycle arrest was observed. Additionally, the therapeutic potential of PRLR in AML was genetically validated.

**Conclusions:**

These results support the role of PRLR as a therapeutic target for AML and the further development of drug discovery programs searching for specific PRLR inhibitors.

**Supplementary Information:**

The online version contains supplementary material available at 10.1186/s12935-023-02944-4.

## Background

The prolactin (PRL) system has emerged as a relevant player in neoplasia, especially in breast and prostate cancers. Although the precise role of PRL-mediated PRL receptor (PRLR) signaling is still controversial in breast cancer cells, PRLR activation seems to be important in tumor initiation, whereas its inhibition may mitigate aggressiveness and/or dissemination of stablished tumors (reviewed in [1]). PRLR is a class I hematopoietic cytokine receptor, that lacks intrinsic enzymatic activity. The Jak2-Stat5 axis is the main PRLR-mediated signal transducer [2]. Contrary to PRL, multiple isoforms of PRLR result from alternative splicing of the primary transcript that mainly affects the intracellular domain and, consequently, the intracellular signaling, although the physiological role of each isoform is still controversial. PRL-induced Stat5 activation is associated to high-grade prostate tumors and aggressiveness, and PRLR inhibition reduced tumor burden [3, 4]. Despite promising preclinical data, the clinical-grade anti-PRLR antibody LFA102 showed no relevant clinical antitumor activity in clinical trials in either tumor type [5]. Overexpression of PRL and/or PRLR were also described in glioblastoma [6], gynecological cancers [7–9], prostate cancer [10] among other tumor types, reinforcing the notion of PRL:PRLR axis as a broad neoplastic feature providing proliferation and/or survival signaling.

Acute Myeloid Leukemia (AML) is a blood neoplasia characterized by the rapid expansion of transformed immature myeloid progenitors in bone marrow and peripheral organs, marked by poor prognosis and frequent relapses. Cytotoxic therapy remains the backbone therapy for the last decades, despite recently approved targeted-pharmacological approaches [11]. Thus, identification of new biomarkers with therapeutic potential is urgently needed. AML cell lines express PRLR, and its activation enhances migration, adhesion and proliferation in vitro [12]. Epidemiologically, hyperprolactinemia is associated with an elevated risk for hematopoietic cancer [13], suggesting that high PRL levels in plasma might correlate with transformation events in the hematopoietic system [14].

Based on the literature and on a preliminary in silico analysis, PRLR was identified as a differentially expressed biomarker in AML cells, at mRNA and surface protein expression levels. Inhibition of this receptor decreased the clonogenicity and engraftment potential. PRLR expression levels directly correlated with resistance to cytarabine, the backbone chemotherapeutics used in AML, and the induction of a senescence-like phenotype. The genetic validation of PRLR as a therapeutic target supports the further development of PRLR-targeted pharmacological approaches for AML.

## Methods

### Primary samples

Primary AML (n = 61) and MDS samples (n = 39) were obtained from patients diagnosed at Hospital Clínic of Barcelona (Spain) and Hospital Germans Trias i Pujol (Badalona, Spain), following the 2016 WHO classification [15] (Table 1 and Additional file 3: Table S1). Healthy-donor buffy coats (n = 7) and umbilical cord blood units (n = 4) were provided by Banc de Sang i Teixits (BST, Barcelona, Spain).

**Table 1:** AML patients’ information

AML	Type	Sex	Age	WHO subtype	BPB (%)	BBM (%)	Karyotype	Additional molecular features	Risk	CR
#1	BM	M	49	Therapy related AML	42	26	46–47, XY, del(5)(q22q34),del(6)(q22q25),del(7)(q22q23),-8,-9,add(11)(q23),+i(11)(q11),-16,+mar1,+mar2,+mar3[cp8]	None	Adv	Yes
#2	PB	M	64	AML without maturation	1	35	46,XY[20]	None	Int	No
#3	N/D	F	45	AML treatment-related	22	44	46,XX[20]	None	Int	No
#4	PB	F	51	AML with mutated *NPM1*	26	63	N/D	NPM1 mut	Fav	No
#5	N/D	M	61	AML with mutated *NPM1*	33	72	46,XY[20]	NPM1 mut, DNMT3A mut	Fav	Yes
#6	N/D	M	51	AML with myelodysplasia-related changes	88	93	46,XY,-5,+mar1[4]/46,XY,-5,+mar2[4]	None	Adv	Yes
#7	N/D	F	54	AML with myelodysplasia-related changes	47	83	48,XX,+add(13)(q34),+add(13)(q34)[8]/46,XX[5]	None	Int	Yes
#8	LA	F	44	AML with mutated *NPM1*	76	80	N/D	NPM1 mut	Fav	No
#9	LA	F	67	MPAL, T/Myeloid, NOS	53	30	46,XX[21]	None	Int	Yes
#10	PB	F	80	AML with mutated *NPM1*	53	95	46,XX[20]	FLT3-ITD, NPM1 mut	Int	No
#11	BM	M	41	AML with t(3;3)(q21.3;q26.2); GATA2. MECOM	57	73	46,XY,t(3;3)(q21;q26)[1]/45,X,-Y,t(3;3)(q21;q26)[19]	None	Adv	Yes
#12	BM	M	45	N/D	N/D	0	46,XY[20]	FLT3-ITD	Adv	No
#13	BM	M	71	AML with mutated *NPM1*	0	3	Normal	NPM1 mut	Fav	No
#14	BM	M	27	N/D	0	2	46,XY[20]	None	Int	No
#15	BM	M	27	AML with myelodysplasia-related changes	3	34	46,XY[37]	FLT3-ITD	Int	No
#16	PB	M	69	AML with myelodysplasia-related changes	23	3	43,XY,-4,add(4)(q?35),del(5)(q13q33),-7,der(12)t(12;?14)(p?12;q?12),-14,-15,del(20)(q11q13),-21,+mar1,+mar2[22]	None	Adv	N/D
#17	BM	F	45	AML with myelodysplasia-related changes	0	20	46,XX[20]	None	Int	No
#18	BM	F	82	AML with mutated *NPM1*	79	83	46,XX,der(12)t(1;12)(q21;q24.3)[4]/46,XX[12]	NPM1 mut	Fav	Yes
#19	BM	M	78	N/D	0	0	Normal	None	Int	No
#20	BM	M	73	N/D	0	27	Complex	None	Adv	No
#21	BM	M	70	N/D	0	5	46,XY[20]	None	Int	No
#22	BM	M	78	N/D	0	0	Normal	None	Int	No
#23	BM	M	27	N/D	0	6	46,XY[20]	None	Int	No
#24	BM	M	47	APL with PML-RARA	71	0	46,XY[20]	N/D	Fav	No
#25	BM	M	71	AML with mutated *NPM1*	N/D	0	Normal	NPM1 mut	Fav	No
#26	BM	M	68	AML with myelodysplasia-related changes	N/D	N/D	N/D	N/D	N/D	N/D
#27	N/D	N/D	N/D	N/D	N/D	N/D	N/D	N/D	N/D	N/D
#28	PB	M	75	N/D	N/D	98	46,XY,inv(3)(p25q21),del(11)(p11.1p15)[cp14]	None	N/D	N/D
#29	BM	M	64	AML with mutated *NPM1*	56	78	46,XY[20]	NPM1 mut	Fav	No
#30	PB	F	47	AML with inv(3)(q21.3q26.2); GATA2, MECOM	3	23	46,XX,inv(3)(q21q26.2)[17]/46,XX[30]	None	Adv	Yes
#31	BM	F	90	AML with myelodysplasia-related changes	92	77	42,XX,del(5)(q22q34),del(7)(q22q32),add(8)(q24),-15,-16,add(17)(p13),-18,-19,-20,-21,-22,-22,+r(?),+mar[cp18]*	TP53 mut	Adv	N/D
#32	PB	F	90	AML with myelodysplasia-related changes	92	77	42,XX,del(5)(q22q34),del(7)(q22q32),add(8)(q24),-15,-16,add(17)(p13),-18,-19,-20,-21,-22,-22,+r(?),+mar[cp18]*	TP53 mut	Adv	N/D
#33	LA	F	63	AML with mutated *NPM1*	100	94	N/D	FLT3-ITD, DNMT3A mut, IDH2mut	Int	No
#34	N/D	N/D	N/D	N/D	N/D	N/D	N/D	N/D	N/D	N/D
#35	N/D	N/D	N/D	N/D	N/D	N/D	N/D	N/D	N/D	N/D
#36	PB	F	64	AML without differentiation	48	73	46,XX[30]	MLL-PTD, IDH2 mut, DNMT3A mut	Adv	No
#37	PB	F	69	AML with mutated *NPM1*	68	66	47,XX,+8[20]	NRAS mut, RUNX1 mut, CEBPA mut	Adv	No
#38	BM	M	62	AML with inv(3)(q21.3q26.2); GATA2, MECOM	18	61	46,XY,inv(3)(q21q26.2)[8]/46,XY[20]	DNMT3A mut, IDH1 mut	Adv	No
#39	PB	F	77	AML with mutated *NPM1*	95	85	N/D	FLT3-ITD, TET2 mut	Int	N/D
#40	PB	M	62	AML with mutated *RUNX1*	49	81	6,XY,del(7)(?)[19]/46,XY[1]	None	Adv	No
#41	N/D	N/D	N/D	N/D	N/D	N/D	N/D	N/D		N/D
#42	PB	M	63	AML with myelodysplasia-related changes	43	30	N/D	TP53 mut	Adv	Yes
#43	BM	M	27	N/D	0	1	46,XY[20]	BCR/ABL absence	Int	No
#44	BM	M	57	N/D	43	33	46,XY[5]	None	Int	No
#45	BM	M	71	AML with mutated *NPM1*	N/D	1	Normal	NPM1 mut	Fav	No
#46	BM	M	73	N/D	0	27	Complex	None	Adv	No
#47	BM	M	43	N/D	29	6	46,XY[30]	None	Int	Yes
#48	BM	M	78	AML with myelodysplasia-related changes	0	N/D	N/D	N/D	N/D	N/D
#49	LA	M	47	AML with mutated *NPM1*	23	48	Normal	DNMT3A mut, IDH2 mut, TP53 mut	Adv	No
#50	LA	M	40	AML with mutated *NPM1*	96	93	N/D	*IDH1* mut, *NPM1* mut, *FLT3*-ITD	Adv	N/D
#51	LA	M	37	AML with inv(16)(p13.1q22); CBFB-MYH11	98	91	47,XY,+9,inv(16)(p13;q22),der(17)t(11;17)(q13;q25)[10]	*CEPBA* mut, *FLT3* mut, *WT1* mut	Fav	No
#52	LA	F	21	AML NOS, monoblastic	98	98	46,XX,t(1;20;11')(p36q21;q11.2;q23),t(10;inv(11)(q13q23))(p12;q13)[20]	KMT2A rearrangement	Adv	No
#53	LA	M	65	AML with mutated *NPM1*	62	80	46,XY[20]	NPM1 mut, FLT3-ITD	Fav	No
#54	PB	M	71	AML with mutated *NPM1*	86	84	N/D	NPM1 mut, FLT3-ITD	Int	N/D
#55	PB	M	55	AML with mutated *NPM1*	85	76	46,XY[20]	NPM1 mut, IDH1 mut, DNMT3A mut, TET2 mut, PTPN11 mut	Fav	N/D
#56	PB	F	68	AML with mutated *NPM1*	54	66	46,XY[20]	NPM1 mut, DNMT3A mut, FLT3-TKD, IDH1 mut, NRAS mut	Fav	No
#57	PB + BM	F	74	AML with biallelic mutation of CEBPA	20	23	46,XX[20]	CEBPA, TET2	Fav	No
#58	PB	F	N/D	N/D	N/D	N/D	N/D	N/D	N/D	N/D
#59	PB	M	84	AML with myelodysplasia-related changes	84	66	46,XY,del(20)(q11q13)[5]/48,XY,+8,del(20)(q11q13),+21[15]	JAK2 mut, IDH2 mut, SRSF2 mut, ETV6-MN1 rearrangement	Int	N/D
#60	PB + BM	M	72	AML with mutated *NPM1*	16	38	46,XY[20]	NPM1 mut, FLT3-ITD, IDH2 mut, RUNX1 mut, WT1 mut, IDH1 mut, DNMT3A mut	Fav	N/D
#61	LA	N/D	N/D	N/D	N/D	N/D	N/D	N/D	N/D	N/D

AML patients’ information

Age in years; *Adv* adverse, *AML* acute myeloid leukemia, *BBM* blasts in bone marrow, *BM* bone marrow, *BPM* blasts in peripheral blood, *CR* chemoresistance, *F* female, *Fav* favorable, *Int* intermediate, *M* male, *N/D* not determined, *PB* peripheral blood, *Risk* cytogenetic risk group according to 2017 ELN recommendations [53], *WHO subtype* WHO subtype according with the 2016 WHO classification [15]

### mRNA expression from public repositories

The analyses from public repositories were mainly performed in the E2: Genomics Analysis and Visualization Platform (https://hgserver1.amc.nl/cgi-bin/r2/main.cgi), using the public database GSE13159 (MILE study) [16, 17]. Probes analysed were: PRLR, 211917_s_at; PRL, 205445_at; Stat3, 208991_at; Stat5a, 203010_at. Diagnosis vs. relapse analysis was performed in the public database GSE66525 [18].

### Cytotoxicity and proliferation assays

1.5 × 10^5^ cells/mL or 2.5 × 10^6^ primary AML cells/mL were cultured in 96-well plates and all drugs (Additional file 2: Methods) were added at the indicated concentrations, as previously described [19]. For proliferation assays, AML cells were stained with 1 µg/mL DiI (Thermo Fisher Scientific) [20].

### Clonogenicity assays

1 × 10^3^ cells of AML cell lines or 50 × 10^3^ primary AML cells or lineage-depleted umbilical cord blood cells were treated at the indicated concentration for 18 h and cultured in 1 mL of MethoCult H4034 Optimum (StemCell Technologies) in 24-well plates, as described previously [19].

### PRLR-silencing with CRISPR/Cas9 technology

Specific sgRNA sequence with the target sequences GAGCCAAGACGCTCACCACTAGG and GTTCGCTGCAAACCAGACCATGG were obtained from ChopChop (https://chopchop.cbu.uib.no/) and cloned into de pLentiGuide-Puro lentiviral vector (Addgene #52963) according to manufacturer’s indications. Cells were co-transduced with pLentiCas9-Blast (Addgene #52962), and selected with puromycin (Sigma-Aldrich) and blasticidin (Sigma-Aldrich). Clones were selected based on the surface expression of PRLR by flow cytometry.

### In vivo studies

6/8 week-old NOD.Cg-*Prkdc*^*scid*^* Il2rg*^*tm1Wjl*^/SzJ (NSG, Jackson Laboratories) mice were myeloablated with intraperitoneal 30 mg/kg busulfan (Sigma-Aldrich). 1 × 10^6^ MonoMac-1 cells transduced with pLL-EF1α-rFLuc-T2A-GFP (rFLuc, System Bioscience #LL410PA-1) were intravenously injected. Leukemia burden was determined by bioluminescence (IVIS Lumina III In vivo Imaging System, Perkin Elmer). Images were analysed with Aura Imaging software (v3.2). For competition assays, an equivalent number of MonoMac-1 cells transduced with pULTRA-Smurf (Addgene #48974) as a control and test-transduced MonoMac-1 were intravenously injected. Engraftment was determined by flow cytometry at day 14.

### Luciferase assays

CISH reporter plasmid was kindly supplied by Dr. Clevenger [21]. pGL4.33[luc2P/SRE/Hygro] (MEK-Erk response element, Promega #E1340); pGL4.29[luc2P/CRE/Hygro] (cAMP response element, Promega #E8471); 4xM67 pTATA TK-Luc (Stat3 response element, Addgene #8688) are commercially available. pRL-SV40 (Addgene #E2231) was used as transfection efficiency control.

### β-Galactosidase staining

5 × 10^5^ cells/mL were attached to poly-l-lysine (50 µg/mL) (Sigma)-coated 96-well plates and stained with the β-Galactosidase Staining Kit (Cell Signal) following the manufacture’s recommendations. Samples were observed at 40× and images were analysed and quantified with ImageJ (v1.8.0_172).

## Results

**Fig. 1:**
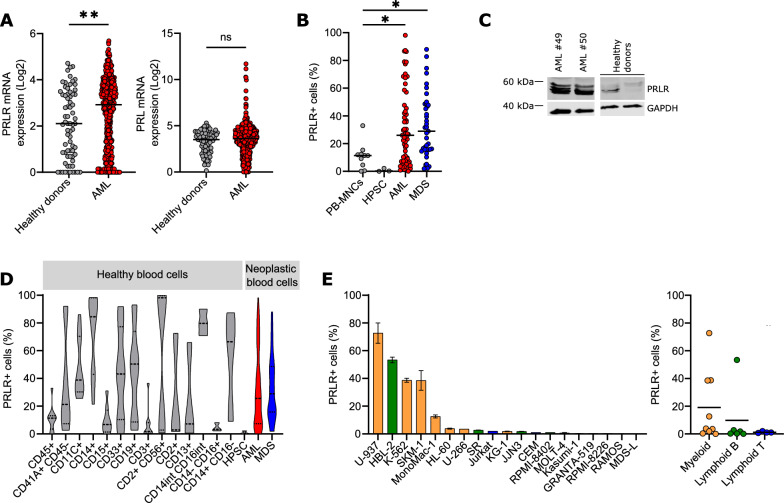
Prolactin receptor is overexpressed in hematological malignancies. **A** PRLR (211917_s_at) and PRL (205445_at) mRNA expression in healthy blood donor cells (grey) and AML cells (red) from the GSE13159 database. Ns, not significant; **p < 0.01 (unpaired t test). **B** PRLR surface expression in CD45^+^ cell population of healthy donors (PB-MNCs n = 10, and HSPC n = 3, grey), AML (n = 59, red), and MDS (n = 39, blue) patient cells were analysed by flow cytometry *p < 0.05 (one-way ANOVA, Dunnett’s multiple comparison test). **C** PRLR protein expression in AML samples (n = 2) and healthy blood donor cells (n = 2) were analysed by Western Blot; GAPDH was used as loading control. **D** PRLR surface expression in different hematopoietic subpopulations of healthy blood cells (n = 10, grey) and in CD45^+^ cell population of neoplastic blood cells [AML (n = 59, red) and MDS (n = 39, blue)] were analysed as in **B**. **E** PRLR surface expression in a panel of cell lines derived from hematological malignancies (n = 19) and classified by phenotype [myeloid (orange), lymphoid B (green) and lymphoid T (blue)] analysed as in **B**. Bars represent the mean ± SEM (duplicates)

As the axis PRL:PRLR regulates key processes in several solid tumors, the role of this signaling pathway was evaluated in the transformation events associated to AML. As a first approach, several AML and healthy blood subpopulations were interrogated for the expression of a PRLR-associated gene signature. The different AML cell fractions clustered together, suggesting that the activation status of this signaling pathway was similar in this population, as compared to healthy blood subpopulations (Additional file 1: Fig. S1A), indicating that AML cells shared a common altered gene signature associated to PRLR. In concordance with this preliminary analysis and previous reports [12], PRLR mRNA was differentially expressed in AML (2.544 ± 0.066) samples as compared to healthy blood donors (2.053 ± 0.174), whereas no significant differences were found in the natural ligand PRL gene expression, at an autocrine level (Fig. 1A). Next, the surface expression of the protein was determined by flow cytometry, as a surrogate of the functional receptor. Similar to the mRNA data, primary patient AML samples expressed higher levels of the PRLR in plasma membrane (Fig. 1B), within the blast gate (SSC-CD45^dim^). Interestingly, not only AML, but also the AML-related myeloid neoplasia Myelodysplastic syndromes (MDS) overexpress PRLR on the cell surface, observing a wide range of expression levels, at a comparable frequency rate (AML: 32.278 ± 3.591%; MDS: 32.947 ± 3.708%). Similar results were obtained at RNA level (Additional file 1: Fig. S1B). PRLR positivity was residual in both healthy blood donor cells sources analysed: peripheral blood mononuclear cells (PB-MNCs) and hematopoietic progenitor/stem cells (lineage-depleted umbilical cord blood cells) (Fig. 1B). Significant differences were also observed at a total PRLR protein level (Fig. 1C). Remarkably, PRLR was expressed on several low-frequent hematopoietic subtypes, such as CD11c-positive dendritic cells or CD14-positive monocytes/macrophages (Fig. 1D). A surface protein expression screening in a wide panel of cell lines derived from different hematological neoplasias revealed that PRLR is expressed on myeloid-associated tumors, mainly AML (U-937, SKM-1, MonoMac-1), while this receptor is generally absent in B or T lymphoid leukemias (Fig. 1E).

**Fig. 2:**
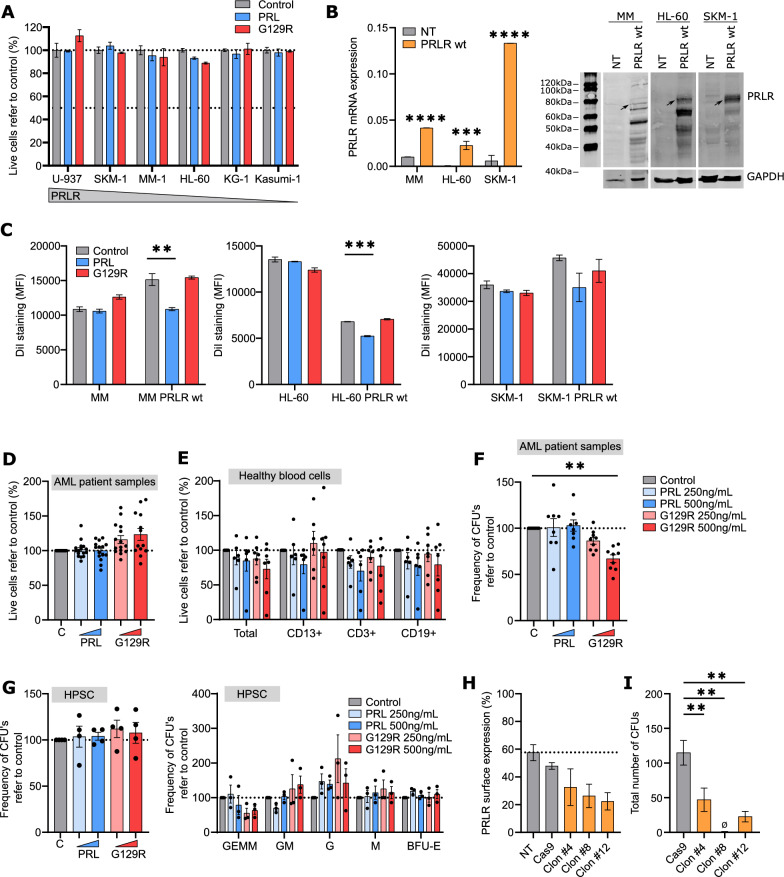
Inhibition of PRLR decreases AML clonogenicity, sparing the healthy counterpart. **A** AML cell lines (HL-60, MonoMac-1, SKM-1, KG-1, U-937, Kasumi-1) (n = 6) were treated with the vehicle (grey), PRL (blue) or G129R (red) at 500 ng/mL for 72 h and cell viability was analysed by flow cytometry (one-way ANOVA, Dunnett’s multiple comparison test, triplicates). **B** PRLR-transduced MonoMac-1 (MM), HL-60 and SKM-1 cells were validated by qPCR (mRNA expression, bars represent 2^−ΔCt^ ± SEM) and Western Blot with GAPDH as loading control (protein, a representative membrane is shown, and arrows showed the interesting band). Grey, control; Orange, PRLRwt-transduced cells. ***p < 0.001; ****p < 0.0001 (two-way ANOVA, Šidák’s multiple comparison test, triplicates). **C** Parental and PRLR-overexpressing MonoMac-1 (MM), HL-60 and SKM-1 cells were treated during 4 days with the vehicle (grey), PRL (blue) or G129R (red) at 500 ng/mL and proliferation (DiI mean fluorescence intensity) was assessed by flow cytometry. **p < 0.01; ***p < 0.001 (two-way ANOVA, Šidák’s multiple comparison test, duplicates). **D** AML patient cells (n = 19) were treated with the vehicle (**C**, grey), PRL (blue) or G129R (red) at 250 and 500 ng/mL and cell viability was assessed after 72 h in the CD45^+^ cell population by flow cytometry. **p < 0.01 (non-parametric one-way ANOVA, Holm-Šidák’s multiple comparison test). **E** Healthy donor PB-MNCs (n = 7) were treated with the vehicle (grey), PRL (blue) or G129R (red) at 250 and 500 ng/mL and cell viability was assessed after 72 h in CD45^+^ total blood population, CD13^+^ myeloid cells, CD3^+^ T cells and CD19^+^ B cells by flow cytometry. Statistics were done as in **D**. **F** AML patient cells (n = 10) were treated with the vehicle (**C**, grey), PRL (blue) or G129R (red) at 250 and 500 ng/mL for 18 h, colonies were counted after 14 days based on cellularity and morphology. Statistics were done as in **D**. **p < 0.01. **G** Lineage-depleted umbilical cord blood cells (n = 4) were treated with the vehicle (grey), PRL (blue) or G129R (red) at 250 or 500 ng/mL for 18 h, colonies were counted after 14 days based on cellularity and morphology. Statistics were done as in **D**. **H** PRLR expression of parental U-937 (grey) and three different PRLR-directed CRISPR/Cas9-transduced clones (orange) were phenotyped for PRLR surface expression by flow cytometry (one-way ANOVA, Dunnett’s multiple comparison test, triplicates). **I** Parental cells (grey) and transduced clones (orange) were cultured in a semisolid medium enriched with cytokines; colonies were counted after 7 days based on cellularity and morphology. **p < 0.01 (one-way ANOVA, Dunnett’s multiple comparison test, n = 6). In all graphs, bars represent the mean ± SEM of each individual experiment

As PRL can act as a survival and proliferation factor [22, 23], several AML cell lines were challenged with the natural ligand PRL and the engineered antagonist del 1-9-G129R-hPRL (G129R) [24]. Cell viability remained unchanged after a 72 h-high dose-treatment with PRL or its antagonist G129R, regardless the PRLR surface expression level (Fig. 2A), as previously demonstrated [12]. As primary AML cells expressed higher levels of PRLR on the surface than AML cell lines (Fig. 1B, E), the long isoform of PRLR (PRLR wt) was ectopically expressed on SKM-1, MonoMac-1 and HL-60 AML cell lines. While SKM-1 expressed intermediate surface levels of endogenous PRLR, MonoMac-1 and HL-60 expressed low levels (Fig. 1E). Interestingly, enforced expression of PRLR in SKM-1 resulted in a notable overexpression of the receptor; while MonoMac-1 and HL-60 presented more modest levels, despite transduction (Fig. 2B and Additional file 1: Fig. S2A). In contrast to the parent cell lines, PRLR wt-overexpressing MonoMac-1 and HL-60 cells responded to the presence of PRL by inducing cell proliferation. However, the antagonist G129R spared AML cell lines in terms of proliferation rate (Fig. 2C). To further explore the effect of PRL in patient cells, primary AML samples (n = 19) from each representative subgroup were treated with PRL and G129R and no significant changes were observed in terms of cell viability after a 72 h treatment (Fig. 2D). Similarly, either PRL or G129R failed to affect survival and/or proliferation of healthy donor total PB-MNCs (n = 7), CD13-positive myeloid, CD3-positive T cells or CD19-positive B cells (Fig. 2E). Since relapse and chemorefractoriness are the main clinical challenges encountered in AML and both properties reside in the most primitive leukemic cell fraction [25, 26], clonogenicity was evaluated upon treatment, as a surrogate assay for stem cell functionality. The potential of primary AML samples (n = 10) to generate blast colonies in a semisolid medium, in the presence of instructive cytokines, remained unaffected in the presence of PRL. However, a 33% reduction was observed in the number of colonies formed when AML samples were treated with the G129R. Interestingly, all tested AML samples were responsive to the treatment (Fig. 2F). In concordance with the lack of effect in cell viability (Fig. 2E), the frequency of both total CFUs and each colony subtype were equivalent in lineage-depleted umbilical cord blood cells (n = 4), regardless of treatment (Fig. 2G). To validate the role of PRLR in clonogenicity, the U-937 AML cells, that express the highest level of this receptor in the surface (Fig. 1E), were edited with a CRIPR/Cas9-based lentiviral system to knock out PRLR up to 2.57-fold (Fig. 2H). In concordance with the results in Fig. 2F, PRLR downregulation resulted in a dramatic decrease of the colony formation capacity (Fig. 2I). Thus, PRLR-mediated signaling seemed to be relevant for self-renewal and differentiation capacities, whereas the importance in survival and proliferation was limited.

**Fig. 3:**
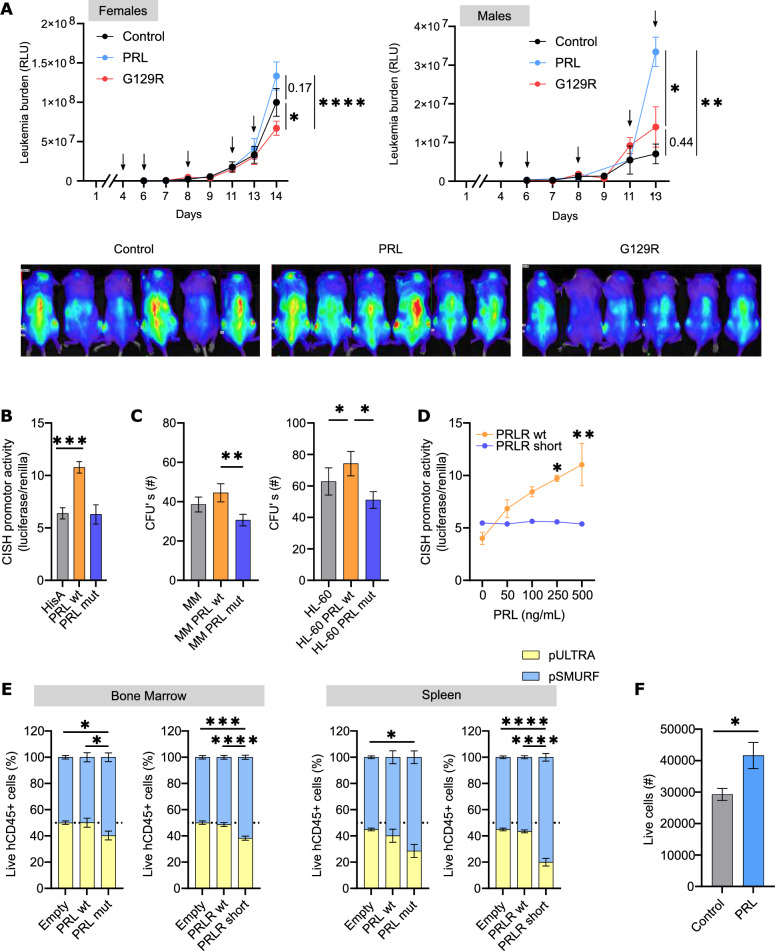
PRLR signaling modulates the in vivo AML regeneration capacity. **A** rFluc-transduced MonoMac-1 (MM) were intravenously injected into adult conditioned NSG mice (n = 34). At day 4, mice were treated 5 days with the vehicle (grey), PRL (blue) or G129R (red) at 0.2 mg/kg. Engraftment was detected by bioluminescence. A representative bioluminescence image at day 13 is presented. *p < 0.05; **p < 0.01; ****p < 0.0001 (one-way ANOVA, Tukey’s multiple comparison test). **B** Activity of the Stat5-reporter CISH was evaluated in the presence of the empty vector (grey), PRL wt (orange) or PRL mut (blue) PRLR-expressing HEK293T cells. ***p < 0.001 (one-way ANOVA, Tukey’s multiple comparison test, triplicates). **C** Parental (grey) and PRL wt- (orange) or PRL mut- (blue) transduced MonoMac-1 (MM, n = 7) and HL-60 (n = 5) were cultured in methylcellulose and colonies were counted after 7 days. *p < 0.05; **p < 0.01 (non-parametric one-way ANOVA, Tukey’s multiple comparison test). **D** Activation of Stat5 reporter CISH was assessed in PRLR wt- (orange) and PRLR short- (blue) transfected HEK293T cells treated with increasing doses of PRL. *p < 0.05; **p < 0.01 (one-way ANOVA, Šidák’s multiple comparison test, triplicates). **E** An equivalent number of pSmurf-transduced MonoMac-1 (blue), and empty control or PRL wt/PRL mut (n = 28) or PRLR wt/PRLR short (n = 31) MonoMac-1 cells (yellow) were intravenously injected into adult conditioned NSG mice. After 15 days, CD45^+^ AML cells from bone marrow and spleen were analysed by flow cytometry. *p < 0.05; ***p < 0.001; ****p < 0.0001 (non-parametric one-way ANOVA, Tukey’s multiple comparison test). **F** Migration capacity was determined using a transwell chamber assays. Growth medium containing 10% FBS was placed in the lower chamber and MonoMac-1 (MM) cells were incubated with the vehicle (grey) or PRL at 500 ng/mL (blue). Cells were allowed to migrate into the low chamber for 48 h. Migrated cells were quantified by flow cytometry. *p < 0.05 (unpaired t test, triplicates). In all graphs, bars represent the mean ± SEM

Next, the role of PRL in a relevant AML in vivo model was evaluated. Adult NSG mice were pharmacologically conditioned and transplanted with rFLuc-AML MonoMac-1 cells. Mice were treated for 5 days with 0.2 mg/kg PRL or G129R, once the engraftment was established. The engraftment levels were monitored by bioluminescence throughout the length of the experiment (schemed in Additional file 1: Fig. S2B). As endogenous murine PRL level in females is greatly increased, compared to males [27], engraftment was analysed within each gender. Moreover, murine PRL not only has more than 50-fold lower potency towards human PRLR than human PRL, but also is an effective competitive inhibitor of human PRL [28]. The treatment with human PRL increased the engraftment level in males; however, no significant differences were observed in females (Fig. 3A), probably due to the differences in murine PRL plasma levels between genders, suggesting that high endogenous mPRL may be disrupting the action of exogenous hPRLR. In concordance, the treatment with the antagonist G129R decreased the engraftment potential of AML, especially in females (Fig. 3A). To limit the effect of the endogenous murine PRL [24] in the xenotransplantation assays, AML cell lines were stably transduced with the wildtype and the mutant G129R PRL (PRL wt and PRL mut, respectively); thus, an autocrine effect was expected without any inter-species interference. As a validation of the system [24], PRL wt induced the transcriptional activation of Stat5-regulated genes (CISH promoter activity), in contrast to PRL mut that failed to induce any transcriptional activity (Fig. 3B). In concordance with the in vivo data (Fig. 3A) and the colony-forming assay (Fig. 2F), PRL mut-expressing AML cells displayed a reduced clonogenic capacity, while PRL wt induced little (if any) differences in terms of blast colony formation (Fig. 3C). Alternative splicing-generated PRLR isoforms differ in the intracellular domain and in their signaling transduction upon activation. The wildtype or long isoform (PRLR wt, 80–85 kDa), predominant in physiological conditions, activates the Jak2/Stat5 pathway upon PRL ligand recognition. In contrast, the short isoform (PRLR short, 45 kDa), whose physiological distribution is controversial, behaves as a dominant negative in some cellular context [29]. Both PRLR wt and PRLR short were transduced into a PRLR^int^ AML cell line, MonoMac-1, to further genetically validate the role of PRLR-mediated signaling in the engraftment potential (Additional file 1: Fig. S2C). As expected, Stat5-mediated transcriptional activation was induced by PRL when PRLR wt was present, in a dose–response manner, and no PRL-induced response was observed in PRLR short (Fig. 3D), confirming the dominant-negative role of the PRLR short in this system. A competitive repopulation assay was chosen to evaluate the relative engraftment potential of the transduced AML cells (GFP-tagged PRLR/PRL isoforms) against the parental leukemic cells (smurf-expressing empty vector). Whereas the leukemic repopulation capacity of AML remained unaffected by the enforced expression of PRL wt or PRLR wt, PRL-neglected signaling AML cells (PRL mut or PRLR short) consistently underperformed, both in bone marrow and spleen (Fig. 3E), supporting the role of PRLR-mediated signaling in the leukemia engraftment potential in vivo. Interestingly, the migration capacity to peripheral organs of both PRL mut- and PRLR short-expressing AML cells was severely diminished, as observed in the leukemia levels in spleen. In concordance, PRL acted as a chemoattractant for AML cells in transwell cell migration assays (Fig. 3F).

**Fig. 4:**
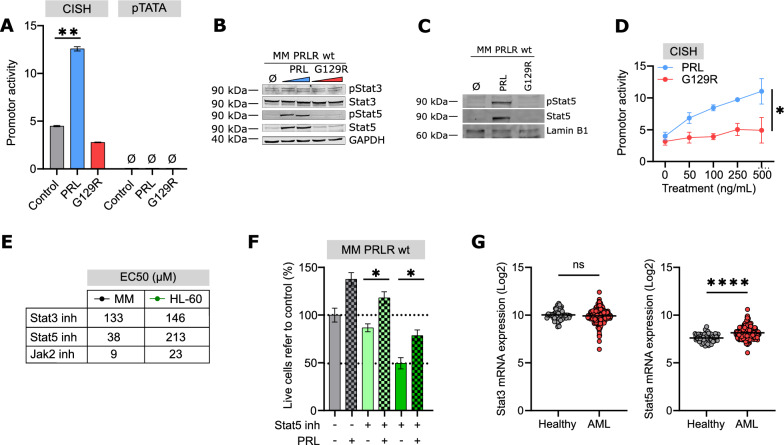
In AML cells, PRLR induces signaling through Jak2/Stat5. **A** CISH and pTATA reporters were transfected in HEK293T cells and treated with the vehicle (grey), PRL (blue) or G129R (red) at 500 ng/mL, in starving conditions. The luciferase activity (normalized against renilla signal) is shown in a representative replicate. **p < 0.01 (two-way ANOVA, Tukey’s multiple comparison test, triplicates). **B** PRLR-transduced MonoMac-1 (MM PRLR wt) cells were treated with the vehicle, PRL (blue) or G129R (red) at 250 and 500 ng/mL for 2 h and total protein were extracted. Total and phosphorylated Stat3 and Stat5 were analysed by Western Blot; GAPDH was used as loading control and a representative membrane is shown (n = 3). **C** PRLR-transduced and control MonoMac-1 (MM) were treated for 2 h with the vehicle, PRL or G129R at 500 ng/mL. Nuclear protein fraction was extracted and total and phosphorylated Stat5 were analysed by Western Blot; Lamin B1 was used as loading control and a representative membrane is shown (n = 3). **D** PRLR wt-expressing HEK293T cells were transfected with the CISH reporter and treated with increasing doses of PRL (blue) or G129R (red) for 24 h. Luciferase activity is shown. *p < 0.05 (paired t test, triplicates). **E** MonoMac-1 and HL-60 cells were treated with an inhibitor of Stat3, Stat5 and Jak2 for 72 h at different doses and viability was analysed by flow cytometry. EC50 value is shown in the table, triplicates. **F** PRLR-transduced and control MonoMac-1 (MM) cells were treated for 72 h with a Stat5 inhibitor (10 and 50 µM) alone (green) or in combination with 500 ng/mL PRL (squared green). Viability was assessed by flow cytometry. *p < 0.05 (one-way ANOVA. Dunnet’s multiple comparison test, triplicates). **G** Stat3 (209991_at) and Stat5 (203010_at) mRNA expression in healthy donors (grey) and AML patients (red) from GSE13159 database. Ns, not significant; ****p < 0.0001 (unpaired t test). In all graphs, bars represent the mean ± SEM

To decipher the signaling pathway associated to PRLR in this cellular context, key response elements were interrogated for their response to PRL. As expected, and unlike Stat3 (pTATA), Jak2/Stat5 is activated upon PRL recognition, inducing Stat5-mediated gene expression (CISH) (Fig. 4A), activating Stat5 by phosphorylation (Fig. 4B and Additional file 1: Fig. S3A) and translocating phospho-Stat5 to the nucleus (Figs. 4C). Despite productive recognition of the PRLR, G129R behaved as an inactive antagonist (Fig. 4D), suggesting that G129R acted as a competitive PRL antagonist [30], rather than as an inverse agonist. Next, the potential regulation of MEK/Erk- (CRE reporter) and cAMP-mediated (SRE reporter) signaling pathways was evaluated and no modulation was observed in response to PRL (Additional file 1: Fig. S3B). In order to explore the dependency of AML cells on the Jak/Stat pathway in the context of the PRLR, the PRLR^int^ AML cell lines MonoMac-1 and the PRLR^low^ AML cell lines HL-60 were treated with specific Stat3 and Stat5 inhibitors. Sensitivity to the Stat3 inhibitor was equivalent in both cell lines (EC50 = 133 µM and 146 µM, respectively) (Fig. 4E), suggesting that PRLR is not involved in this signaling pathway, in concordance with the lack of transcriptional activity upon PRL activation (Fig. 4A). The cytotoxic effect induced by the inhibition of Jak2 was also similar in both AML cell lines (Fig. 4E), discarding a major contribution of Jak2 to the PRLR-mediated signaling. In contrast, a lower EC50 was observed in PRLR^int^ AML cells (MonoMac-1), as compared to PRLR^low^ AML cells (HL-60), when challenged with the Stat5 (30 µM vs. 213 µM) inhibitor (Fig. 4E and Additional file 1: Fig. S3C). Thus, the sensitivity to the Stat5 inhibitor was related to the PRLR expression levels. Indeed, the cytotoxicity induced by the Stat5 inhibitor was partially reverted in the presence of PRL (Fig. 4F), in AML cell lines, suggesting that Stat5-activated signaling pathway was regulated by PRL. Moreover, Stat5 mRNA was overexpressed in AML patient samples (GSE13159 [16, 17]), as compared to healthy donors, while Stat3 mRNA levels are equivalent in both populations (Fig. 4G), highlighting the pivotal role of Stat5 in the PRLR-mediated leukemic features.

**Fig. 5:**
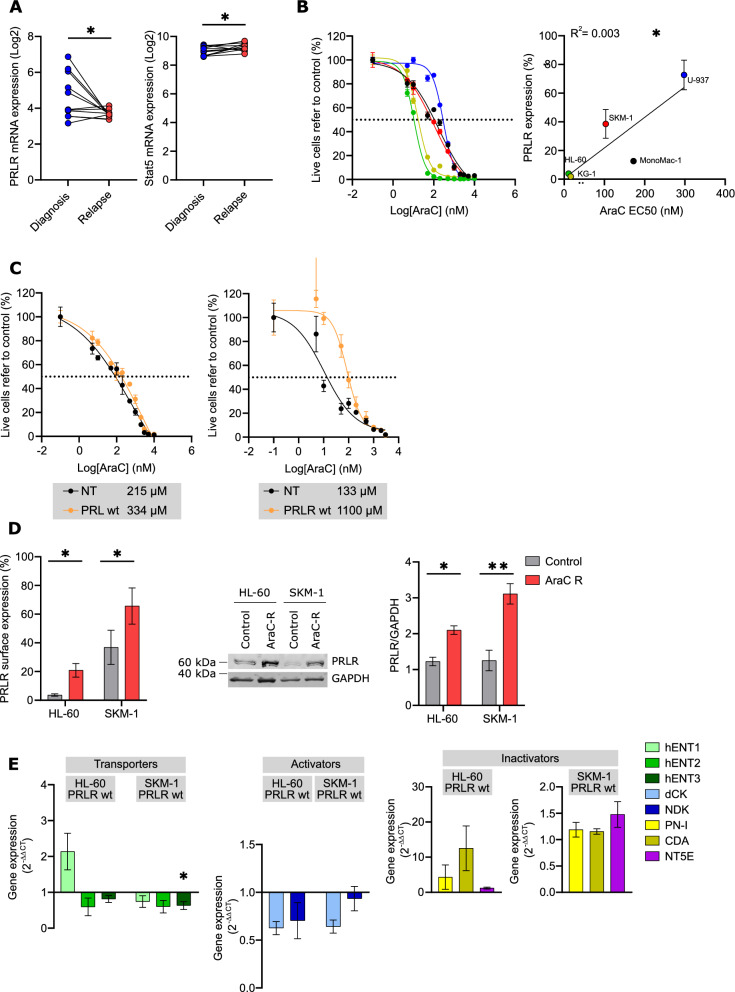
PRLR confers chemoresistance to AML. **A** PRLR and Stat5a mRNA expression at diagnosis (blue) and relapse (red) in AML patients from GSE66525 database. *p < 0.05 (unpaired t test). **B** AML cell lines were treated for 72 h with cytarabine (AraC) at the indicated doses. Viability was assessed by flow cytometry and a representative result was shown. The EC50 values were correlated with the PRLR surface expression analysed by flow cytometry. *p < 0.05 (simple linear regression, triplicates). Green, HL-60; Red, SKM-1; Blue, U-937; Black, MonoMac-1 (MM); Brown, KG-1. **C** PRLR-transduced (orange), PRL-transduced (orange) and control (black) MonoMac-1 (MM) cells were treated with AraC for 72 h. Viability was analysed by flow cytometry and representative results were shown (n = 3). **D** PRLR expression in AraC-resistant (AraC R, red) and the parental (grey) cell lines HL-60 and SKM-1 were analysed by flow cytometry (protein surface expression) and western blot (total protein level). *p < 0.05; **p < 0.01 (paired t test, triplicates). **E** Expression of genes associted to cytarabine transporters (green), activators (blue) and inactivators (yellow and purple) were determined by qPCR in PRLR wt-tranduced HL-60 and SKM-1 cells and compared to parental cells. *p < 0.05 (one-way ANOVA, Dunnett’s multiple comparison test, triplicates). In all graphs, bars represent the mean ± SEM

To evaluate the role of PRLR signaling during leukemogenesis, paired diagnosis-relapse AML samples were analyzed for their PRLR and Stat5 mRNA levels to track the expression of the receptor and the main secondary messenger through the course of the disease. Stat5 gene expression increased in relapse disease, although the expression of PRLR mRNA decreased (Fig. 5A); however, surface expression of the receptor was similar at diagnosis and relapse in a limited patient cohort (Additional file 1: Fig. S4A). Interestingly, sensitivity to cytarabine, the backbone of most chemotherapeutic regimens for AML, correlated with PRLR surface expression. The highest the endogenous expression of PRLR corresponded to the highest resistance to cytarabine treatment (Fig. 5B). While enforced expression of PRL in AML cells failed to confer chemoresistance to cytarabine, overexpression of PRLR significantly augmented the EC50 of cytarabine in AML cells (Fig. 5C), suggesting that the PRLR-mediated signaling was responsible for overcoming the cytotoxicity effect produced by cytarabine. Besides, acquired resistance to cytarabine in AML cell lines by long-term drug treatment (Additional file 1: Fig. S4B) resulted in the upregulation of PRLR at surface and total protein expression levels (Fig. 5D). At a gene expression level, overexpression of PRLR induced the downregulation of key cytarabine transporters (*hENT2* and *hENT3*) and cytarabine activators (*dCK* and *NDK*), while inactivators such as *PN-I*, *CDA* and *NT5E* were upregulated (Fig. 5E). Thus, the expression of PRLR induced a chemoresistance-associated gene signature [31].

**Fig. 6:**
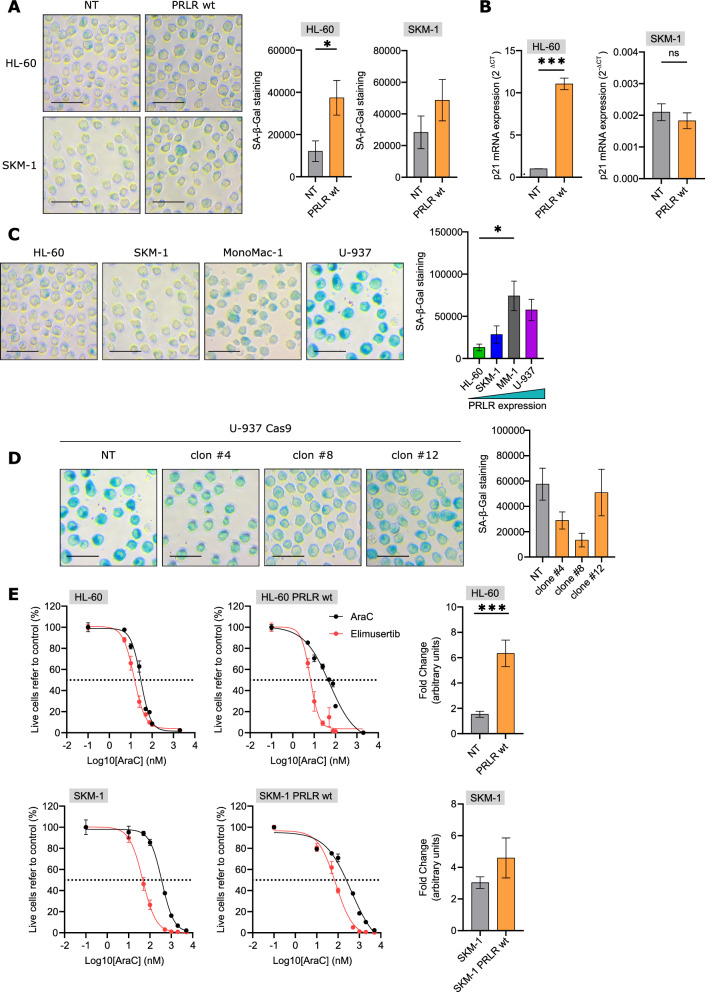
PRLR induces a senescence-like phenotype that is associated with chemoresistance. **A** SA-β-galactosidase staining in PRLR wt-tranduced (orange) and parental control (grey) HL-60 and SKM-1. *p < 0.05 (unpaired t test, n = 4). **B** p21 mRNA expression in PRLR-tranduced (orange) and parental control (grey) HL-60 and SKM-1 cells analysed by qPCR. Bars represented 2^ΔCt^ ± SEM of three independent experiments. ***p < 0.001 (unpaired t test). **C** SA-β-galactosidase staining in HL-60 (n = 4, green), SKM-1 (n = 4, blue), MonoMac-1 (MM, n = 6, grey) and U-937 (n = 3, purple) cells. *p < 0.05 (one-way ANOVA, Tukey’s multiple comparison test). **D** SA-β-galactosidase staining in CRISPR/Cas9-tranduced (orange) and parental control (grey) U-937 clones (n = 3, one-way ANOVA, Dunnett’s multiple comparison test). **E** PRLR wt-transduced (orange) and parental control (grey) HL-60 and SKM-1 cells were treated for 48 h with increasing cytarabine doses alone (AraC, black) or in combination with 20 nM elimusertib (red). Viability was analysed by flow cytometry and representative results were graphicated. EC50 value was calculated (left), as well as the fold change in the EC50 (1-(EC50 AraC/cotreatment EC50) (n = 4, right); ***p < 0.001 (unpaired t test). In all graphs, bars represent the mean ± SEM

Recently, chemoresistant AML cells have been associated with a senescence-like phenotype, maintaining the leukemia repopulation potential [25]. To explore the senescence phenotype in relationship to PRLR expression, X-gal-based β-galactosidase staining was examined as a canonical biomarker for this state [32]. As demonstrated by the increase of senescence-associated β-gal (SA-β-gal) staining (Fig. 6A), PRLR overexpression induced a senescence-like phenotype; although no consistent differences were observed in the proliferation rate (Additional file 1: Fig. S5A), p21 or p16 upregulation (Fig. 6B and Additional file 1: Fig. S5B), or cell cycle status (Additional file 1: Fig. S5C), precluding cell cycle arrest [33], and suggesting the acquisition of a premature senescence-like phenotype. Interestingly, basal SA-β-gal staining was associated with PRLR expression (Fig. 6C). These findings were further validated using a CRISPR/Cas9-based strategy to downregulate the expression of PRLR (Fig. 2H), reducing the SA-β-gal positivity (Fig. 6D). The highest the expression of PRLR, the highest the resistance to cytarabine and the highest the senescence marker staining. In AML, induction of senescence as well as tumor survival and chemotherapy persistence are dependent on ATR activity. Previous studies showed that ATR inhibition synergized with nucleoside analogues, like cytarabine, to eradicate AML in xenotransplantation mouse models [34]. Indeed, an ATR-dependent senescence-like resilient state was demonstrated to be responsible for AML relapse, at least partially. This resilient state had a variable duration and conferred an optimized fitness and enhanced repopulation capacity [25]. In concordance, the presence of elimusertib, an ATR inhibitor, increased the sensitivity to PRLR-overexpressing AML cells to cytarabine treatment, as compared to the parental cell line (Fig. 6E), suggesting that ATR played a key role in the chemoresistance observed upon enforced expression of PRLR wt.

## Discussion

Refractory and relapsed (R/R) AML is still highly challenging from a clinical point of view, as poor response rates and, consequently, unfavorable prognosis are expected. Given the need for more effective treatment options in R/R AML, improving the efficacy of salvage chemotherapy by combining targeted therapy or optimizing drug delivery had been explored with limited success [11]. Despite inconsistencies regarding the response rates for most of the investigational agents [hypomethylating agents (decitabine or azacitidine) [35], Bcl-2 inhibitor (venetoclax) [35], tyrosine kinase inhibitors (sorafenib [36], pazopanib [37], quizartinib [38] or crenolanib [39]), or MAPK (binimetinib [40] or trametinib [41]) and hedgehog (glasdegib) [42] modulators] and combinations, durable responses were achieved in certain patients; though it will be important to identify biomarkers that predict individual patient response. Nevertheless, disruptive therapeutic approaches are urgently needed as the chemotherapy sphere might have reached its limits and the therapeutic window of most targeted therapies is fairly narrow.

Senescence, chemoresistance and cancer stemness are closely interrelated. A senescence-like phenotype is observed in resistant AML cells upon chemotherapy [25, 26, 43], associated with an increase in colony-forming and engraftment potential [25]. After recovery, these cells are enriched in leukemic stem cells [25, 44], linking the senescence program to the acquisition of cancer stemness properties. PRLR overexpression correlated to both the senescence-like phenotype, as measured by SA-β-Gal staining, and the resistance to cytarabine. Disruption of the PRLR signaling also affected leukemic stemness, as measured in clonogenic assays. Similar to the chemoresistance-induced senescence resilient phenotype where ATR catalytic activity enables the induction of this cellular state, tumor survival and persistence [25], ATR was also involved in the PRLR-mediated resistance to cytarabine. Interestingly, PRLR-induced senescence-like phenotype failed to induce cell arrest, as proliferation was not affected, in concordance with previous reports [25, 44].

Personalized medicine requires the identification of new functional biomarkers, such as PRLR. Expression of PRLR was described in a limited cohort (9 patients) of monoblast-like M4 AML patients several decades ago [45] and autocrine/paracrine PRL was associated with survival/proliferation of AML cells [46]. From an epidemiology standpoint, leukemia is the second most frequent cancer associated to hyperprolactinemia in females [13]. Here, the therapeutic potential of PRLR in AML is corroborated, and associated with Stat5 activation and chemoresistance phenotypes. Abnormal activation of Stat5 via phosphorylation is frequently observed in AML cells [47], induces proliferation of leukemic cells [48] and desensitizes to certain drugs [49]. A *STAT5*-associated signature correlated to unfavorable clinical outcome in AML patients [50]. Indeed, pharmacological inhibition of Stat5 reduced leukemia burden in an in vivo FLT3-ITD + AML model [51]. Although promising, no clinical-grade Stat5 small molecule inhibitors have been proven in clinical trials. Clinical experiences from Stat3 inhibitors suggested low specificity and wide side effects [52], probably due to a poor therapeutic window based on the pleiotropic effect of Stat3. As PRLR is differentially expressed and activated in leukemic cells, targeting directly this receptor might provide a safe therapeutic window to further explore this approach in clinical development. LFA102, a clinical-grade PRLR neutralizing antibody, failed to provide any clinical benefit in breast and prostate cancer patients [5], although insufficient dose exposure and inhibition efficacy in vivo might account for these negative results. Despite the dedication of great efforts from the scientific community in developing small molecules with antagonistic effect on PRLR, and based on the key role of this receptor in leukemogenesis, designing novel therapeutic strategies to specifically disrupt the PRLR-mediated signaling in leukemic cells might provide a promising approach to overcome relapse and refractory episodes in AML.

## Conclusions

- Prolactin Receptor is differentially expressed in AML and its expression positively correlates with chemoresistance to cytarabine.
- Inhibition of the PRLR-mediated signaling reduces the clonogenicity and the engraftment potential of AML in xenotransplantation mouse models.

## Supplementary Information

**Additional file 1: Figure S1.** PRLR signaling pathway is differentially expressed in AML in comparison to HSCs and early precursors. **A** mRNA expression profiles related to PRLR signalingwere selected and hierarchy grouped without supervision, previous normalization by RMA. AML patient samples enrich in LSCs were marked. **B** PRLRmRNA expression in healthy blood donor cells, ALL, AML, CLL, CML, and MDSfrom the GSR13159 database. **p < 0.01; ***p < 0.001; ****p < 0.0001.. **Figure S2.** Validation of PRLR-transduced cells and AML murine model. **A** PRLR surface expression in PRLR wt-transducedand parental controlMonoMac-1, HL-60 and SKM-1 cells by flow cytometry. A representative histogram is shown. **B** rFLuc-transduced MonoMac-1cells were intravenously injected into adult conditioned NSG mice. At day 4, 6, 8, 11 and 13 mice were treated intraperitoneally with the vehicle, PRL or G129R at 0.2 mg/kg. Engraftment was followed by bioluminescence at day 4, 6, 8, 11, 13 and 14. **C** PRLR wt-and PRLR short-transducedMonoMac-1cells were validated by qPCRand Western Blotwith GAPDH as loading control and a representative membrane is shown. *p < 0.05; ****p < 0.0001. **Figure S3.** PRLR signals through Jak2/Stat5. **A** PRLR-transduced MonoMac-1 cellswere treated with the vehicle, PRLor G129Rat different doses for 2 h and total protein lysates were obtained. Phosphorylated and total Stat5 were analysed by Western Blot; GAPDH was used as loading control. A representative membrane is shown. **B** CRE and SRE reporters were transfected in HEK293T cells and treated with the vehicle, PRLor G129Rat 500 ng/mL. The luciferase activity is shown in a representative replicate. **p < 0.01. **C** MonoMac-1and HL-60were treated with a Stat3, Stat5 and Jak2 inhibitors for 72 h at different doses and viability was analysed by flow cytometry. A representative result was shown. Bars represent the mean ± SEM. **Figure S4.** PRLR confers chemoresistance to AML. **A** PRLR surface expression analysis in non-refractoryvs. refractoryAML patient samplesby flow cytometry. **B** Cytarabine-resistantand the parentalcell lines HL-60 and SKM-1were validated by flow cytometry. A representative result is shown. **Figure S5.** PRLR overexpression does not affect AML proliferation capacity. **A** Proliferationwas assessed in PRLR-transducedand parental controlHL-60 and SKM-1 cells by flow cytometry. **B** p16 mRNA expression in PRLR-tranducedand parental controlHL-60 and SKM-1 cells analysed by qPCR. **C** Cell cycle analysis of PRLR-transduced and parental control HL-60 and SKM-1 analysed by flow cytometry. A representative histogram is shown. **Figure S6.** A graphical summary of the role of PRLR-PRL signaling in AML.

**Additional file 2.** Methods.

**Additional file 3: Table S1.** MDS patients’ information.

## Data Availability

For original data or detailed protocols, please contact Dr. Risueño risueno@carrerasresearch.org.

## Abbreviations

AML: Acute myeloid leukemia
PB-MNC: Peripheral blood mononuclear cell
PLR: Prolactin
PRLR: Prolactin receptor
