# Supplementary material for: Prolactin receptor signaling induces acquisition of chemoresistance and reduces clonogenicity in acute myeloid leukemia
Source: Cancer Cell Int. 2023 May 19;23:97. doi: 10.1186/s12935-023-02944-4 (PMC10197460; doi:10.1186/s12935-023-02944-4)
Supplement: Supplementary file 1 — Additional file 1: Figure S1. PRLR signaling pathway is differentially expressed in AML in comparison to HSCs and early precursors. A mRNA expression profiles related to PRLR signalingwere selected and hierarchy grouped without supervision, previous normalization by RMA. AML patient samples enrich in LSCs were marked. B PRLRmRNA expression in healthy blood donor cells, ALL, AML, CLL, CML, and MDSfrom the GSR13159 database. **p < 0.01; ***p < 0.001; ****p < 0.0001.. Figure S2. Validation of PRLR-transduced cells and AML murine model. A PRLR surface expression in PRLR wt-transducedand parental controlMonoMac-1, HL-60 and SKM-1 cells by flow cytometry. A representative histogram is shown. B rFLuc-transduced MonoMac-1cells were intravenously injected into adult conditioned NSG mice. At day 4, 6, 8, 11 and 13 mice were treated intraperitoneally with the vehicle, PRL or G129R at 0.2 mg/kg. Engraftment was followed by bioluminescence at day 4, 6, 8, 11, 13 and 14. C PRLR wt-and PRLR short-transducedMonoMac-1cells were validated by qPCRand Western Blotwith GAPDH as loading control and a representative membrane is shown. *p < 0.05; ****p < 0.0001. Figure S3. PRLR signals through Jak2/Stat5. A PRLR-transduced MonoMac-1 cellswere treated with the vehicle, PRLor G129Rat different doses for 2 h and total protein lysates were obtained. Phosphorylated and total Stat5 were analysed by Western Blot; GAPDH was used as loading control. A representative membrane is shown. B CRE and SRE reporters were transfected in HEK293T cells and treated with the vehicle, PRLor G129Rat 500 ng/mL. The luciferase activity is shown in a representative replicate. **p < 0.01. C MonoMac-1and HL-60were treated with a Stat3, Stat5 and Jak2 inhibitors for 72 h at different doses and viability was analysed by flow cytometry. A representative result was shown. Bars represent the mean ± SEM. Figure S4. PRLR confers chemoresistance to AML. A PRLR surface expression analysis in non-refractoryvs. refractoryAML pat [file 12935_2023_2944_MOESM1_ESM.docx]

**Additional Figures**

**Figure S1**

**
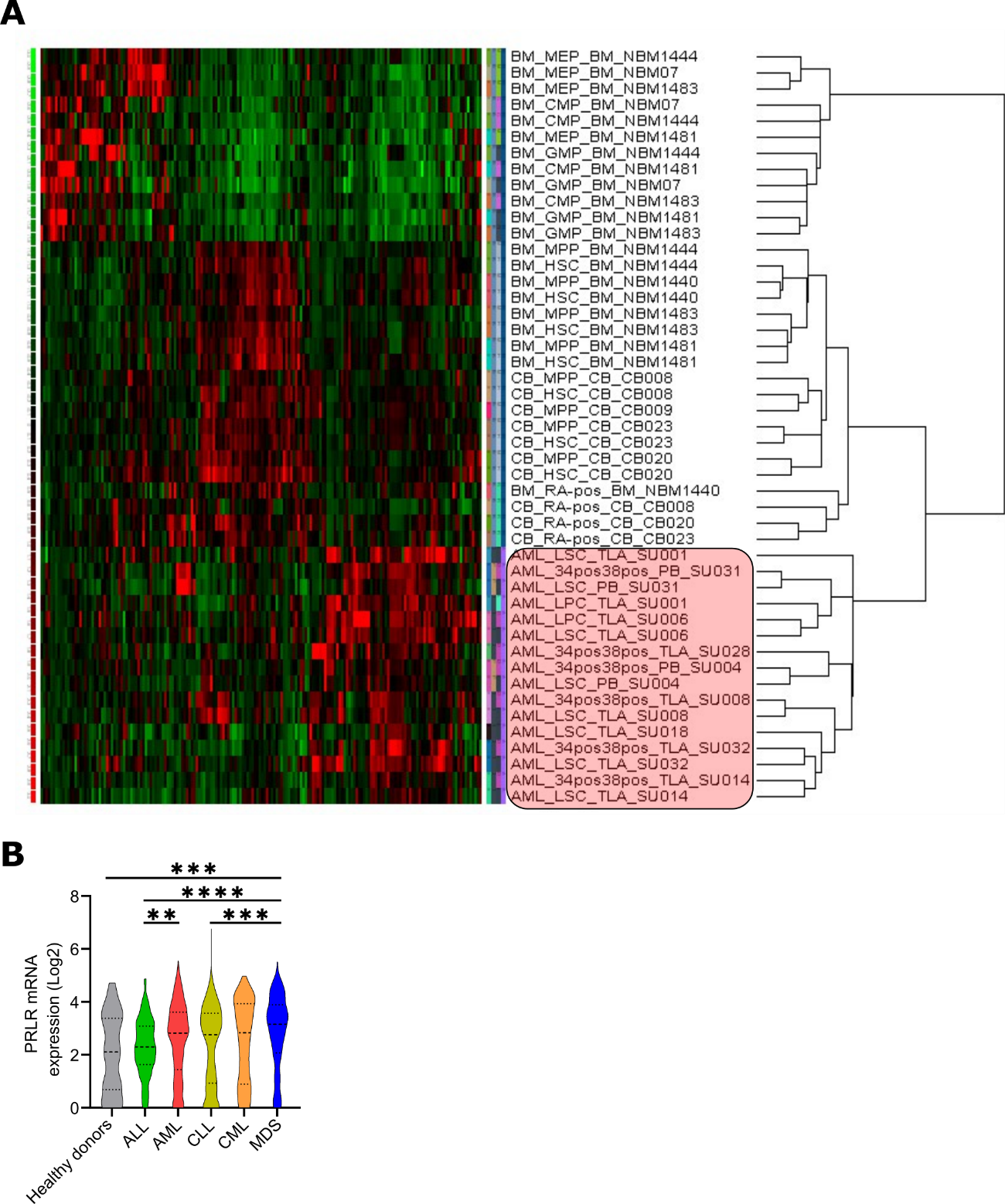
**

**Figure S1. PRLR signaling pathway is differentially expressed in AML in comparison to HSCs and early precursors. A)** mRNA expression profiles related to PRLR signaling (GSE24006 and KEGG Prolactin signaling pathway hsa04917) were selected and hierarchy grouped without supervision, previous normalization by RMA (Robust Multichip Average). AML patient samples enrich in LSCs were marked. **B)** PRLR (211917_s_at) mRNA expression in healthy blood donor cells (grey), ALL (acute lymphoblastic leukemia, green), AML (acute myeloid leukemia, red), CLL (chronic lymphocytic leukemia, yellow), CML (chronic myeloid leukemia, orange), and MDS (myelodysplastic syndromes, blue) from the GSR13159 database. **p<0.01; ***p<0.001; ****p<0.0001. (One-way ANOVA, Tukey’s multiple comparison test).

**Figure S2**

**
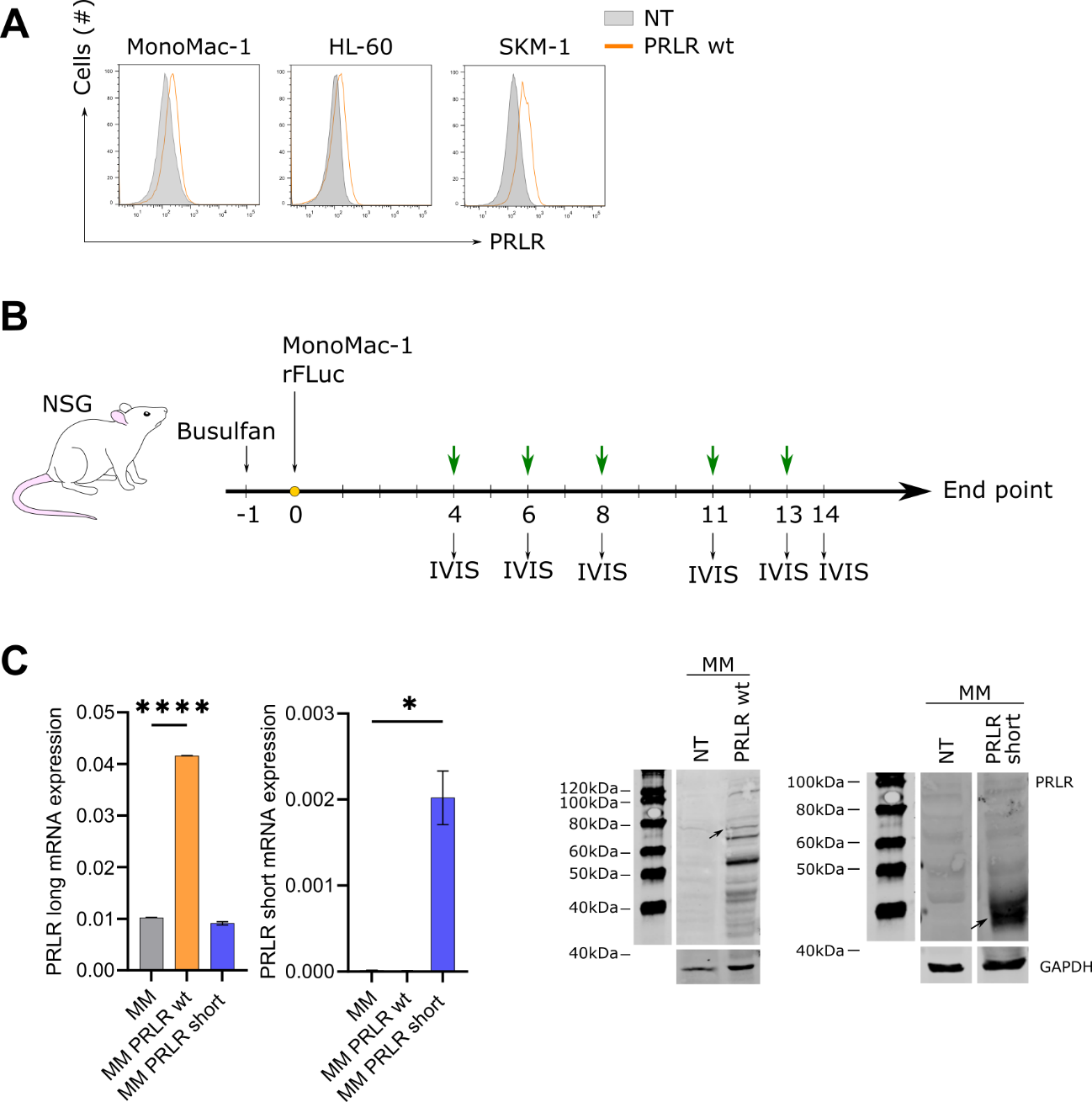
**

**Figure S2. Validation of PRLR-transduced cells and AML murine model. A)** PRLR surface expression in PRLR wt-transduced (orange) and parental control (grey) MonoMac-1 (MM), HL-60 and SKM-1 cells by flow cytometry. A representative histogram is shown (n=3). **B)** rFLuc-transduced MonoMac-1 (MM rFluc) cells were intravenously injected into adult conditioned NSG mice. At day 4, 6, 8, 11 and 13 mice were treated intraperitoneally with the vehicle, PRL or G129R at 0.2 mg/kg. Engraftment was followed by bioluminescence at day 4, 6, 8, 11, 13 and 14. **C)** PRLR wt- (orange) and PRLR short-transduced (blue) MonoMac-1 (MM) cells were validated by qPCR (mRNA expression, bars represent 2^-ΔCt^ ± SEM) and Western Blot (protein) with GAPDH as loading control and a representative membrane is shown. *p<0.05; ****p<0.0001 (two-way ANOVA, Šidák’s multiple comparison test, triplicates).

**Figure S3**

**
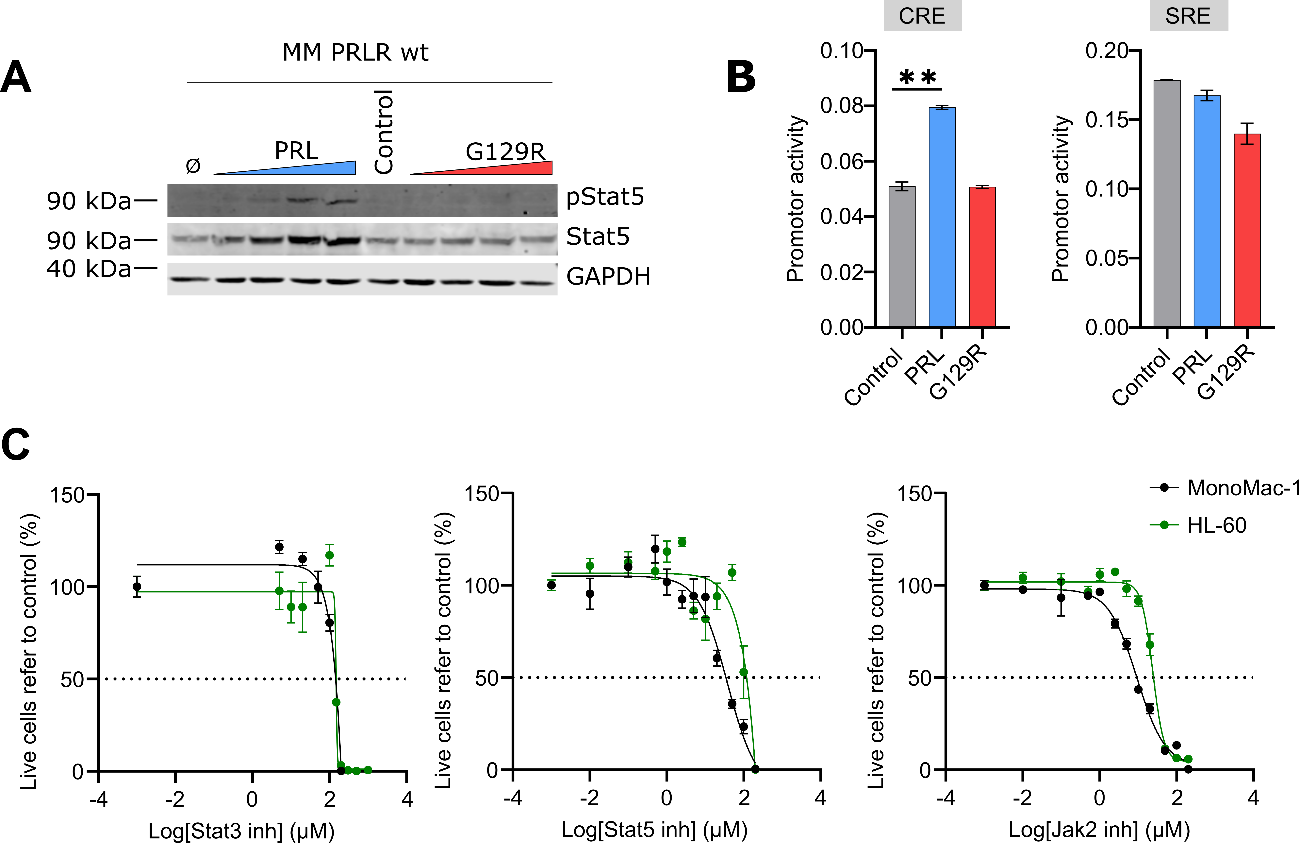
**

**Figure S3. PRLR signals through Jak2/Stat5. A)** PRLR-transduced MonoMac-1 cells (MM PRLR wt) were treated with the vehicle, PRL (blue) or G129R (red) at different doses for 2h and total protein lysates were obtained. Phosphorylated and total Stat5 were analysed by Western Blot; GAPDH was used as loading control. A representative membrane is shown (n=3). **B)** CRE and SRE reporters were transfected in HEK293T cells and treated with the vehicle (grey), PRL (blue) or G129R (red) at 500 ng/mL. The luciferase activity is shown in a representative replicate. **p<0.01 (two-way ANOVA, Tukey’s multiple comparison test, triplicates). **C)** MonoMac-1 (MM, black) and HL-60 (green) were treated with a Stat3, Stat5 and Jak2 inhibitors for 72h at different doses and viability was analysed by flow cytometry. A representative result was shown (n=3). Bars represent the mean ± SEM.

**Figure S4**

**
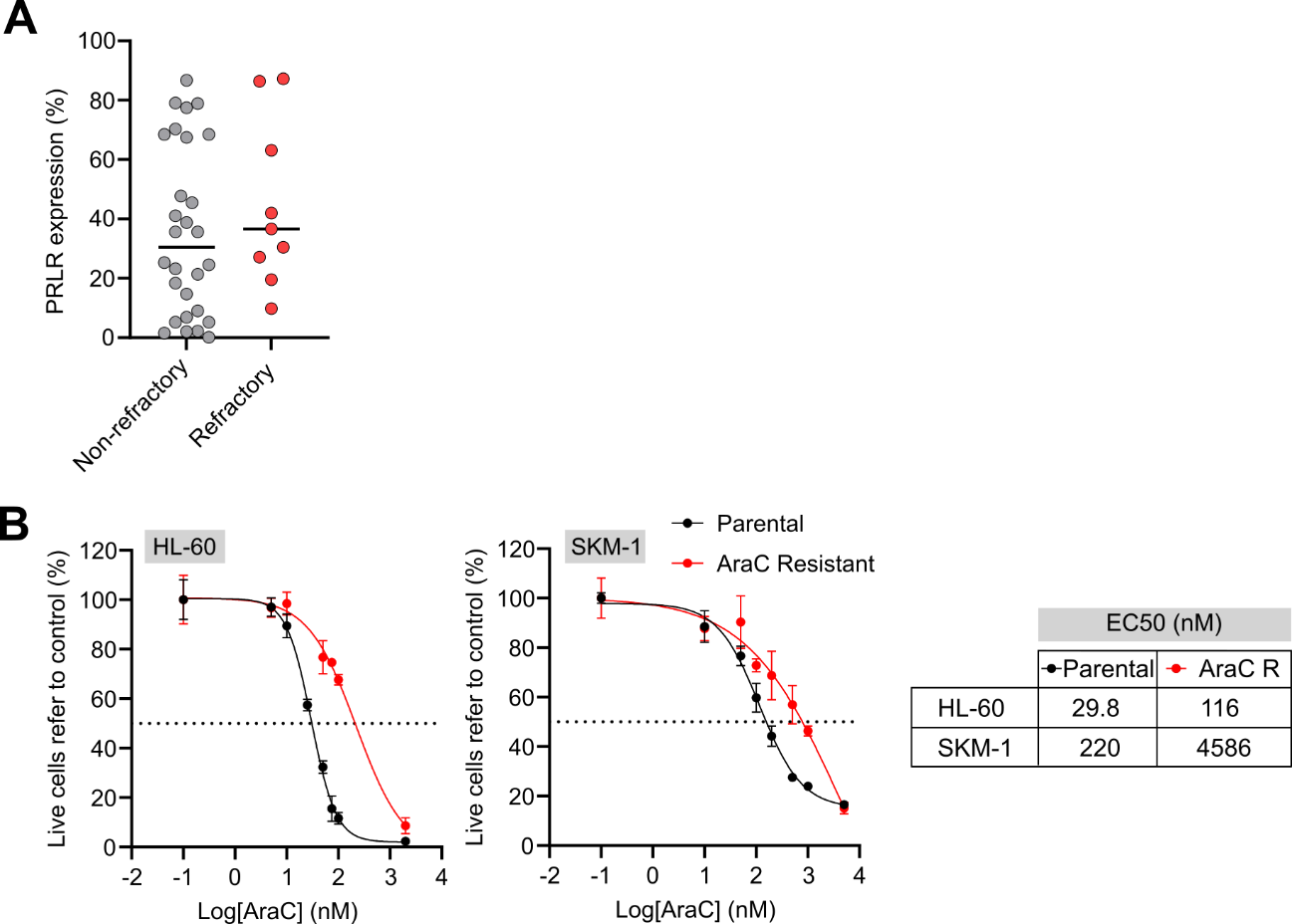
**

**Figure S4. PRLR confers chemoresistance to AML. A)** PRLR surface expression analysis in non-refractory (grey) vs. refractory (red) AML patient samples (n=59) by flow cytometry (unpaired t test). **B)** Cytarabine-resistant (red, AraC resistant) and the parental (black) cell lines HL-60 and SKM-1 (black) were validated by flow cytometry. A representative result is shown (n=3).

**Figure S5**

**
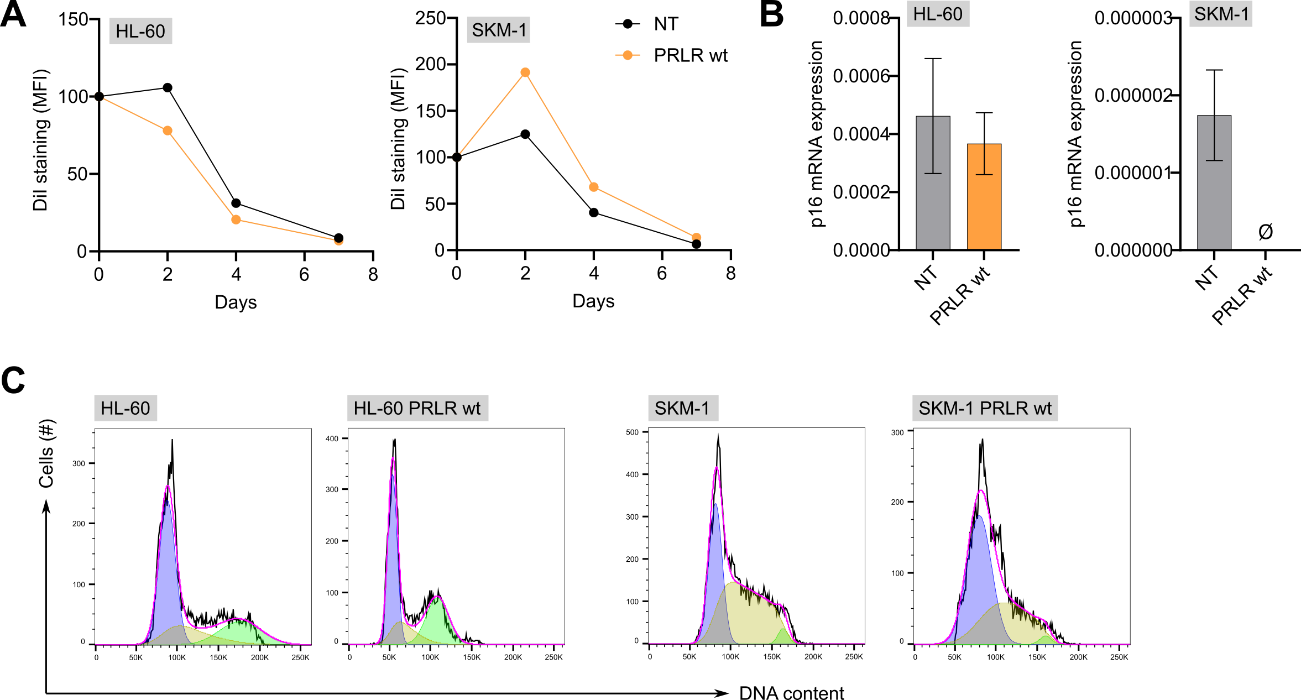
**

**Figure S5. PRLR overexpression does not affect AML proliferation capacity. A)** Proliferation (DiI mean fluorescence intensity) was assessed in PRLR-transduced (orange) and parental control (black) HL-60 and SKM-1 cells by flow cytometry (paired t test, duplicates). **B)** p16 mRNA expression in PRLR-tranduced (orange) and parental control (grey) HL-60 and SKM-1 cells analysed by qPCR (Unpaired t test, triplicates, bars represented 2^-ΔCt^ ± SEM). **C)** Cell cycle analysis of PRLR-transduced and parental control HL-60 and SKM-1 analysed by flow cytometry. A representative histogram is shown (n=2).

**Figure S6**

**
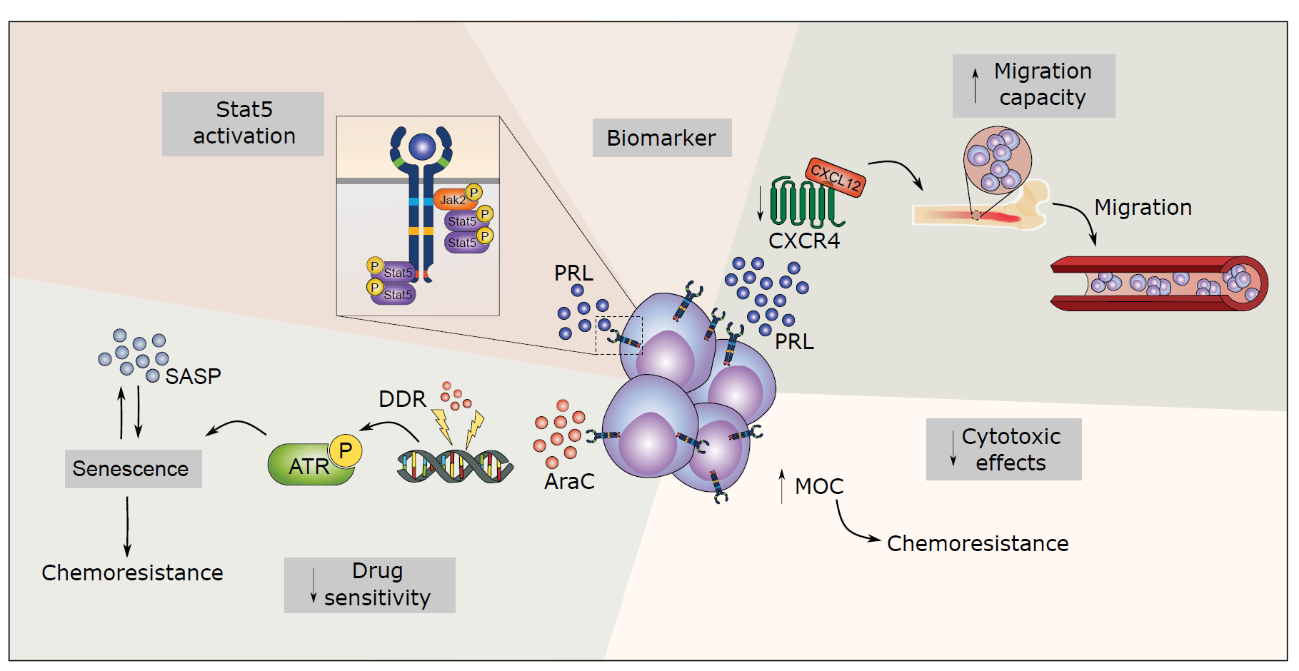
**

**Figure S6.** A graphical summary of the role of PRLR-PRL signaling in AML.
