# Supplementary material for: Prolactin receptor signaling induces acquisition of chemoresistance and reduces clonogenicity in acute myeloid leukemia
Source: Cancer Cell Int. 2023 May 19;23:97. doi: 10.1186/s12935-023-02944-4 (PMC10197460; doi:10.1186/s12935-023-02944-4)
Supplement: Supplementary file 2 — Additional file 2. Methods. [file 12935_2023_2944_MOESM2_ESM.docx]

**METHODS**

**Gene expression analysis**

Gene expression profiles corresponding to primary AML-LSC (Lin^-^CD34^+^CD38^-^CD90^-^) and HSCs from both CB and BM (Lin^-^CD34^+^CD38^-^CD90^+^CD45RA^-^) were obtained from the public repository GEO with accession number GSE24006. Array data were normalized, and comparisons were performed using DNA-Chip Analyzer (dChip) Software (Harvard School of Public Health, Boston, MA, USA). dChip Software implements invariant set normalization and probe-level model-based expression analysis on multiple arrays and computes the t-statistic and the *P*-value based on the *t*-distribution. Hierarchical clustering was performed with dChip, where the distance between two genes was defined as 1–*r*, where *r* is the Pearson's correlation coefficient. Sample clustering was performed based on expression values of genes related to PRLR signaling (GSE24006 and KEGG Prolactin signaling pathway hsa04917) ^1^.

**Cell cultures**

Cell lines U-937 (ACC-5), K-562 (ACC-10), SKM-1 (ACC-547), MonoMac-1 (ACC-252), HL-60 (ACC-3), U-266 (ACC-9), KG-1 (ACC-14), JJN-3 (ACC-541), CCRF-CEM (ACC-240), RPMI-8402 (ACC-290), MOLT-4 (ACC-362), Kasumi-1 (ACC-220), GRANTA-519 (ACC-342), RPMI-8226 (ACC-402), RAMOS (ACC-603), and HEK-293T (ACC-635) were obtained from DSMZ. SR (CRL-2262^TM^) and HEK-293T (CRL-3216^TM^) cell lines were obtained from ATCC. HBL-2 cell line was kindly supplied by Dr. Pérez-Galán (IDIBAPS, Barcelona, Spain), Jurkat cell line by Dr. Bigas (IMIM, Barcelona, Spain), MDS-L cell line by Dr. Starczynowki (CCHMC, Cincinnati, US) ^2^, and SKM-1 and U-937 cell lines by Dr. Buschbeck (IJC, Badalona, Spain). Cell lines were cultured in complete RPMI1640 (AML cell lines, Biowest) or DMEM (HEK-293T, Biowest). Primary AML blasts were cultured in complete IMDM (Biowest), as described previously ^3^. AML and MDS patient's characteristics are summarized in Table 1. Mononuclear cells (MNCs) were isolated by Ficoll density gradient centrifugation (GE). Samples were cryopreserved in cryopreservation media (90% HI-FBS, 10% DMSO) and store in liquid nitrogen. Phenotype analysis was performed either in freshly isolated mononuclear cells or in recently thaw samples. All patients provided written informed consent in accordance with the Declaration of Helsinki, and the study was approved by the corresponding Ethics Committees. Blood MNCs were isolated from healthy-donor buffy coats. MNCs from umbilical cord bloods were depleted for lineage marker-positive cells using magnetic separation, following manufacturer's recommendations (Miltenyi Biotec).

**Drugs**

All drugs were resuspended in H_2_O (Thermo Fischer Scientific) or DMSO (Sigma-Aldrich) according to manufacturer’s specifications.

**Table 1. Chemical compounds used.**

| **Name** | **Vendor** | **Action** | **CAS num** | **Solvent** |
| --- | --- | --- | --- | --- |
| Prolactin (PRL) | PreProtech | PRLR agonist | - | H_2_O |
| del 1-9-G129R (G129R) | MyBiosource | PRLR antagonist | - | H_2_O |
| Stat3 inhibitor | Sigma-Aldrich | pStat3 inhibition | 22112-89-6 | DMSO |
| Stat5 inhibitor | Sigma-Aldrich | pStat5 inhibition | 285986-31-4 | DMSO |
| Ruxolitinib phosphate (Ruxo) | Signalway antibody | Jak2 inhibition | 941678-49-5 | DMSO |
| Cytarabine (AraC) | Sigma-Aldrich | Antileukemic | 147-94-4 | H_2_O |
| Elimusertib (BAY-1895344, Elimu) | Selleckchem | ATR inhibitor | 1876467-74 | DMSO |

**Surface phenotype**

Cells were stained in surface with the indicated antibody. In malignant samples, the analysis was performed inside the blast gate (CD45^dim^SSC^int^). In healthy samples, PRLR expression analysis was performed in each cell subset with the indicated markers. Samples were acquired in a FACSCanto II cytometer (BD), and results were analysed using FlowJo software (v10.7.2).

**Table 2. Antibodies used for flow cytometry**

| **Antibody** | **Clon** | **Vendor** | **Fluorochrome** |
| --- | --- | --- | --- |
| CD11b/Mac-1 | ICRF44 | BD | PE |
| CD14 | M5E2 | BD | APC |
|  | MΦP9 | BD | V450 |
| CD45 | HI30 | BD | FITC, PE, APC, V450 |
| CD41a | HIP8 | BD | FITC |
| CD11c | B-ly6 | BD | APC |
| CD33 | WM53 | AB D serotec | Alexa488 |
|  | HIM3-4 | BD | FITC, PE |
| CD13 | WM15 | BD | PE |
|  |  | AB D serotec | Alexa647 |
| CD15 | HI98 | BD | APC |
| CD19 | SJ25C1 | BD | FITC |
| CD3 | HIT3a | BD | APC |
| CD2 | RPA-2.10 | BD | FITC |
| CD56 | B159 | BD | PE |
| CD16 | B73.1 | BD | APC |
| CD34 | 581 | BD | APC, PE |
| CD38 | HIT2 | BD | FITC, PE |
| CD45 (mouse) | 30-F11 | BD | PE |

**Cytotoxicity and proliferation assays**

1.5 x 10^5^ cell lines/mL or 2.5 x 10^6^ primary AML cells/mL were cultured in 96-well plates in complete medium. Drugs (specified above) were added at the indicated concentrations. Cell viability was measured by 7-AAD (eBioscience) exclusion by flow cytometry and cell count was obtained by volume in a FACSCantoII cytometer (BD). FlowJo software (TriStar) was used for flow cytometry analysis.

A total of 3 × 105 cells per ml were cultured in 24-well plates in complete medium. Cells were stained with 1 μM Vybrant DiI Cell-Labeling Solution (Invitrogen), a liphophilic membrane stain. Every two days cells were acquired in a FACSCanto II (Becton Dickinson) and FCS files were analyzed in the FlowJo software (Tristar).

**Clonogenicity**

A total of 50 × 103 primary AML cells or 1 × 103 cells from AML cell lines were treated at the indicated concentration for 18 h, and cultured in 1 ml of MethoCult H4034 Optimum (StemCell Technologies). Colonies were screened based on morphology and cellularity at day 14 (primary AML cells) or 7 (AML cell lines).

**Protein detection**

1x10^6^ AML cells or 25-50x10^6^ primary AML or healthy donor cells were lysed in RIPA lysis buffer for the total protein extraction or using *NE-PER Nuclear and Cytoplasmatic Extraction Reagents^TM^* (Thermo Fischer Scientific) for the nuclear fraction extraction according to manufacturer’s instructions. Protein detection was performed by Western Blot. Antibodies used for this technique are listed on the table below. Images were acquired and analysed in *Odyssey Infrared Imaging System®* (LI-COR).

**Table 3. List of antibodies used for Western Blot and Flow Cytometry.**

| **Antibody** | **Clon** | **Reference** | **Vendor** |
| --- | --- | --- | --- |
| PRLR | 1A2B1 | #32-9200 | Invitrogen |
| PRLR | U5 | MA1-610 | Thermo Fischer Scientific |
| GAPDH | 6C5 | AM4300 | Thermo Fisher Scientific |
| pStat3 | 3E2 | #9132 | Cell Signaling |
| Stat3 | 79D7 | #4904 | Cell Signaling |
| pStat5 | 8-5-2 | #9351 | Cell Signaling |
| Stat5 | Policlonal | ab126832 | Abcam |
| Lamin B1 | B-10 | sc-374015 | Santa Cruz Biotechnology |

**Real Time qPCR**

Total RNA were isolated using the *Total RNA Purification Kit* (Norgen Biotek) and reversely transcribed using *qScript cDNA Synthesis Kit* (Quanta Bioscience), and the following manufacturers’ recommendations. Primer pairs are summarized in Table 4. Results are presented as Fold Changes normalized to GAPDH (2^-ΔCt^) or together with the control cells (2^-ΔΔCt^).

**Table 4. List of primers used for RT-qPCR.**

| Amplified gene | Sense | Sequence |
| --- | --- | --- |
| GAPDH | Fw | GTGGACCTGACCTGCCGTCT |
|  | Rv | GGAGGAGTGGGTGTCGCTGT |
| PRLR long isoform | Fw | TCCAGGTATGTGGGTTTCAT |
|  | Rv | GATTTGATGCTCATCTGTTGGA |
| hENT1 | Fw | GGGCAGCCTGTTTGGTCT |
|  | Rv | CCACTGGCAATAGCGCAG |
| hENT2 | Fw | CTCCTGTCCATGGCCAGTG |
|  | Rv | GGGCCTGGGATGATTTATTG |
| hENT3 | Fw | TCAGCGGTGCCTCCACTGT |
|  | Rv | GCAGCCAAGTCCACCAATGA |
| dCK | Fw | GCCGCCACAAGACTAAGGAA |
|  | Rv | GACTTCCCTGCAGCGATGTT |
| NDK | Fw | ATTCCGCCTTGTTGGTCTGA |
|  | Rv | TTGGAGTCTGCAGGGTTGGT |
| PN-I | Fw | AACAACATAGCATCCCCGTGT |
|  | Rv | TTCCTCAAGGCACCATCATGT |
| CDA | Fw | TGTGCTGAACGGACCGCTA |
|  | Rv | GCAGGTCCTCAGGCCCAA |
| NT5E | Fw | TCTTCTAAACAGCAGCATTCC |
|  | Rv | CATTTCATCCGTGTGTCTCAG |
| p16 | Fw | CCAACGCACCGAATAGTTACG |
|  | Rv | GCGCTGCCCATCATCATG |
| p21 | Fw | TGGAGACTCTCAGGGTCGAAA |
|  | Rv | GGCGTTTGGAGTGGTAGAAATC |

**Generation of PRL and PRLR overexpressing cells**

To express PRL, wild-type PRL (PRL wt) was amplified by PCR from MonoMac-1 gDNA (forward primer, 5’-CCCGATATCCCACTAGTATATGAACATCAAA-3’; reverse primer, 5’-CCCGATATCTTGGATCCAATTAGCAGTTGTTGT-3’) using KOD DNA Polymerase (Millipore) according to manufacturer’s instructions. Inactive PRL form (PRL mut) were achieved by site-directed mutagenesis of PRL wt (Gly129Arg) ^4^. Both PRL wt and PRL mut were cloned in the intermediate vector pEF.1 (Invitrogen #V92020) and in the lentiviral vector pULTRA (Addgene #24129) ^5^. To overexpress PRLR different isoforms, the intermediate plasmid pEF.1 with PRLR wt insert was kindly supplied by Dr. Clevenger (Virginia Commonwealth University, EEUU) ^6^, while PRLR short was subcloned in the same intermediate vector pEF.1 (forward primer, 5’-CCCGAATTCATAATGAAGGAAAATGTGG-3’; reverse primer, 5’-CCCTCTAGAATAAGGGGTCACCTCCAACAG-3’). Both isoforms were also cloned in the lentiviral vector pULTRA.

**Migration assays**

Migration capacity was determined using transwell chamber assays. 2.5 x 10^5^ cells/mL were culture in 24-well plates with the indicated treatment and 5% FBS and allowed to migrate to a lower chamber with 20% FBS as chemoattractant for 48h. Migrated cells were quantified by flow cytometry.

**Cytarabine-resistant cell lines**

HL-60 and SKM-1 were treated with cytarabine at the EC50 value until the tissue culture recovered. Then, the cytarabine dose were increased up to duplicate the EC50 value.

**Cell cycle analysis**

5x10^5^ cells/mL were cultured in 6-well plates in starving conditions for 18h and then cultured in complete medium for 24h. Cells were harvested, washed, and fixed and permeabilized in 70% ethanol (Sigma-Aldrich) at 4ºC. DNA content was stained with Hoechst33342 (Sigma-Aldrich) and measured by flow cytometry.

**Ethics approval**

All patients provided written informed consent in accordance with the Declaration of Helsinki, and the study was approved by the corresponding Ethics Committees (Ethics Committee Hospital Clínic de Barcelona, Ethics Committee Hospital Germans Trias i Pujol). All experiments involving mice were approved by the Catalan Ethical Committee of Animal Experimentation (CCEEA).

**Statistical analysis**

Statistical significance was determined using GraphPad Prism® 8.0 (GraphPad software) by using statistical tests specified in figure legends. Normality tests were done for the experiments with AML cell lines and its Gaussian distribution was determined to perform bilateral and non-paired Student t tests with 95% of confidence. ANOVA tests were also performed. Gaussian distribution was not assumed when AML primary samples were analysed and bilateral non-parametric Mann-Whitney tests with a 95% confidence were performed. Errors bars correspond to SEM. All experiments were done at least 3 times in biological triplicates, otherwise it is specified in the figure legend.

**REFERENCES**

1. Etxabe A, Lara-Castillo MC, Cornet-Masana JM, et al. Inhibition of serotonin receptor type 1 in acute myeloid leukemia impairs leukemia stem cell functionality: a promising novel therapeutic target. *Leukemia*. 2017;31(11):2288–2302.

2. Matsuoka A, Tochigi A, Kishimoto M, et al. Lenalidomide induces cell death in an MDS-derived cell line with deletion of chromosome 5q by inhibition of cytokinesis. *Leukemia*. 2010;24(4):748–755.

3. Moreno-Martínez D, Nomdedeu M, Lara-Castillo MC, et al. XIAP inhibitors induce differentiation and impair clonogenic capacity of acute myeloid leukemia stem cells. *Oncotarget*. 2014;5(12):4337–4346.

4. Goffin V, Kinet S, Ferrag F, et al. Antagonistic properties of human prolactin analogs that show paradoxical agonistic activity in the Nb2 bioassay. *J. Biol. Chem.* 1996;271(28):16573–16579.

5. Lou E, Fujisawa S, Morozov A, et al. Tunneling nanotubes provide a unique conduit for intercellular transfer of cellular contents in human malignant pleural mesothelioma. *PLoS One*. 2012;7(3):.

6. Kline JB, Moore DJ, Clevenger C V. Activation and association of the Tec tyrosine kinase with the human prolactin receptor: Mapping of a Tec/Vav1-receptor binding site. *Mol. Endocrinol.* 2001;15(5):832–841.
