# Supplementary material for: Prolactin receptor signaling induces acquisition of chemoresistance and reduces clonogenicity in acute myeloid leukemia
Source: Cancer Cell Int. 2023 May 19;23:97. doi: 10.1186/s12935-023-02944-4 (PMC10197460; doi:10.1186/s12935-023-02944-4)
Supplement: Supplementary file 3 — Additional file 3: Table S1. MDS patients’ information. [file 12935_2023_2944_MOESM3_ESM.docx]

**Table S1. MDS patients’ information.** Abbreviations: 2016 WHO classification ^1^; BBM: blast in bone marrow; F: female; IPSS-R: Revised International Prognostic Scoring System ^2^; M: male; MDS: myelodysplastic syndromes; MDS del(5q): MDS with isolated 5q deletion; MDS-EB-1: MDS with excess of blasts type 1; MDS-EB-2: MDS with excess of blasts type 2; MDS-MLD: MDS with multiple linage dysplasia; MDS-RS: MDS with ring sideroblats; MDS-RS-MLD: MDS with ring sideroblasts and multiple linage dysplasia; MDS-RS-SLD: MDS with ring sideroblasts and single linage dysplasia; N/D: not determined.

| **MDS** | **Age (years)** | **Sex** | **2016 WHO classification** | **Karyotype** | **IPSS-R** | **BBM (%)** |
| --- | --- | --- | --- | --- | --- | --- |
| #1 | 75 | M | MDS-MLD | 46,XY[20] | Low | 0 |
| #2 | 83 | M | MDS-EB-1 | 47,XY,+8[3]/46,XY[28] | High | 7 |
| #3 | 46 | F | MDS-RS | 46,XX[20] | Low | 2 |
| #4 | 70 | F | MDS-RS | 46,XX[20] | Intermediate | 4 |
| #5 | 74 | M | MDS-EB-2 | 45,X,-Y,del(1)(p13p32),der(11)t(Y;11)(q11;q13)[8]/46,XY[12] | Very high | 12 |
| #6 | 75 | M | MDS-MLD | 46,XY[20] | Low | 0 |
| #7 | 73 | F | MDS del(5q) | 46,XX,del(5)(q22q33)[15]/46,XX[5] | Low | 2 |
| #8 | 51 | M | MDS-MLD | N/A (normal FISH 5p15.2, 5q31, 7q31 & 20q12) | N/A | 2 |
| #9 | 71 | F | N/D | 46 XX,-11,+mar [20] | Intermediate | 3 |
| #10 | 29 | F | MDS del(5q) | 46,XX,del(5)(q12q32)[11]/46,XX[9] | N/D | N/D |
| #11 | 81 | F | MDS-MLD | 46,XX[10] | Low | 0 |
| #12 | 59 | F | MDS del(5q) | 46,XX,del(5)(q13q33)[4]/46,XX[15] | Low | 2 |
| #13 | 65 | M | MDS-RS-MLD | 46,XY[20] | Very low | 2 |
| #14 | 86 | F | MDS-MLD | 47,XX,+8[10]/48,idem,+mar[2]/46,XX[7] | High | 3 |
| #15 | 65 | M | MDS-RS-MLD | 46,XY[20] | Very low | 2 |
| #16 | 64 | M | MDS del(5q) | 46,XX,del(5)(q14q34)[5]/46,XX[15] | Very low | 1 |
| #17 | 78 | M | MDS del(5q) | 46,XY,del(5)(q13q33)[12]/46,XY[8] | Very low | 2 |
| #18 | 77 | M | MDS-EB-2 | Pérdida 3p, deleción 5, pérdida 17p. Inv(9) constitucional | Very high | >5% |
| #19 | 67 | M | MDS-MLD | 46,XY[20] | Low | 4 |
| #20 | 83 | M | MDS-RS-SLD | 46,X,-Y,+8[1]/45,X,-Y[5]/46,XY[13] | Intermediate | 1 |
| #21 | 60 | F | MDS-RS-MLD | 46,XX[20] | Very low | 1 |
| #22 | 91 | F | MDS-EB-1 | 45,X,-Y[14]/46,XY[6] | High | 7 |
| #23 | 67 | M | MDS-EB-2 | 43,XY,-5,der(13;14)(q10;q10),add(15)(p10),add(16)(q24),-17,add(17)(p13),-18,+mar[cp14] | Very high | 15 |
| #25 | 79 | M | MDS-EB-1 | 46,XY[21] | High | 5 |
| #26 | N/D | N/D | N/D | N/D | N/D | N/D |
| #27 | 94 | F | MDS del(5q) | 46,XX,del(5)(q13q33)[9]/46,XX[24] | Very low | 1 |
| #28 | N/D | N/D | N/D | N/D | N/D | N/D |
| #29 | N/D | N/D | N/D | N/D | N/D | N/D |
| #30 | 68 | F | MDS-MLD | 46,XX[20] | Very low | 0 |
| #31 | 83 | M | MDS-MLD | 45,X,-Y[18]/46,XY[2] | Low | 3 |
| #32 | N/D | N/D | N/D | N/D | N/D | N/D |
| #33 | 58 | M | MDS-SLD | 46,XY,inv(2)(p23q13),del(5)(q13q32) | Low | 1 |
| #34 | N/D | N/D | N/D | N/D | N/D | N/D |
| #35 | 68 | F | MDS-MLD | 46,XX,del(9)(q22)[11]/46,XX[2] | Intermediate | 0 |
| #36 | 75 | F | MDS-MLD | 46,XX,-5,-14,+mar1,+mar2[16]/45,XX,del(5)(q13q33),-6[2]/46,XX[8] | High | 1 |
| #37 | 83 | F | MDS-EB-1 | del(7)(q22q31) | Very high | 5 |
| #38 | 30 | F | MDS-MLD | 46,XX[20] | Very low | 2 |
| #39 | 66 | M | MDS-EB-1 | 47,XY,+8[2]/46,XY[3] | Intermediate | 5 |
